# Cost-Effectiveness of Cervical Cancer Screening in Women Living With HIV in South Africa: A Mathematical Modeling Study

**DOI:** 10.1097/QAI.0000000000001778

**Published:** 2018-06-15

**Authors:** Nicole G. Campos, Naomi Lince-Deroche, Carla J. Chibwesha, Cynthia Firnhaber, Jennifer S. Smith, Pam Michelow, Gesine Meyer-Rath, Lise Jamieson, Suzette Jordaan, Monisha Sharma, Catherine Regan, Stephen Sy, Gui Liu, Vivien Tsu, Jose Jeronimo, Jane J. Kim

**Affiliations:** * Harvard T.H. Chan School of Public Health, Department of Health Policy and Management, Boston, MA;; † Health Economics and Epidemiology Research Office, Department of Internal Medicine, School of Clinical Medicine, Faculty of Health Sciences, University of the Witwatersrand, Johannesburg, South Africa;; ‡ Division of Global Women's Health, University of North Carolina at Chapel Hill, Chapel Hill, NC;; § Clinical HIV Research Unit, Helen Joseph Hospital, Johannesburg, South Africa;; ║ Right to Care, Helen Joseph Hospital, Johannesburg, South Africa;; ¶ Department of Epidemiology, Gillings School of Global Public Health, University of North Carolina, Chapel Hill, NC;; # National Health Laboratory Service, Johannesburg, South Africa;; ** Anatomical Pathology Department, Faculty of Health Sciences, University of the Witwatersrand, Johannesburg, South Africa;; †† Department of Global Health and Development, School of Public Health, Boston University, Boston, MA;; ‡‡ Department of Epidemiology, University of Washington, Seattle, WA;; §§ PATH, Seattle, WA; and; ║║ Global Coalition Against Cervical Cancer, Arlington, VA.

**Keywords:** HIV, cancer screening, HPV, HPV DNA test, cervical cytology, South Africa, mathematical model, cost-effectiveness

## Abstract

**Background:**

Women with HIV face an increased risk of human papillomavirus (HPV) acquisition and persistence, cervical intraepithelial neoplasia, and invasive cervical cancer. Our objective was to determine the cost-effectiveness of different cervical cancer screening strategies among women with HIV in South Africa.

**Methods:**

We modified a mathematical model of HPV infection and cervical disease to reflect coinfection with HIV. The model was calibrated to epidemiologic data from HIV-infected women in South Africa. Clinical and economic data were drawn from in-country data sources. The model was used to project reductions in the lifetime risk of cervical cancer and incremental cost-effectiveness ratios (ICERs) of Pap and HPV DNA screening and management algorithms beginning at HIV diagnosis, at 1-, 2-, or 3-year intervals. Strategies with an ICER below South Africa's 2016 per capita gross domestic product (US$5270) were considered “cost-effective.”

**Results:**

HPV testing followed by treatment (test-and-treat) at 2-year intervals was the most effective strategy that was also cost-effective, reducing lifetime cancer risk by 56.6% with an ICER of US$3010 per year of life saved. Other cost-effective strategies included Pap (referral threshold: HSIL+) at 1-, 2-, and 3-year intervals, and HPV test-and-treat at 3-year intervals. Pap (ASCUS+), HPV testing with 16/18 genotyping, and HPV testing with Pap or visual triage of HPV-positive women were less effective and more costly than alternatives.

**Conclusions:**

Considering per capita gross domestic product as the benchmark for cost-effectiveness, HPV test-and-treat is optimal in South Africa. At lower cost-effectiveness benchmarks, Pap (HSIL+) would be optimal.

## Abstract 2

Supplemental Digital Content is Available in the Text.

## INTRODUCTION

Compared with the general population, women living with HIV face an increased risk of acquiring human papillomavirus (HPV), the sexually transmitted virus that causes cervical cancer.^1^ Once infected with HPV, women with HIV face heightened risk of HPV persistence and precancerous lesions, which may progress to invasive cervical cancer if not detected through screening and effectively treated.^2^ Among women with HIV in South Africa, where HIV prevalence is 22.3% in those aged 15–49 years,^3^ the age-standardized incidence rate of cervical cancer is estimated to be 396 per 100,000 person-years^4^—more than 10 times higher than the rate in the general population.^5^ As access to antiretroviral therapy (ART) has improved, with more than 60% of HIV-infected adults expected to be receiving ART,^6^ life expectancy in women who begin ART with a CD4^+^ cell count above 200 cells/µL has increased dramatically.^7^ Organized cervical cancer screening efforts are needed to ensure that potential life expectancy gains from ART are fully realized among women with HIV.

The World Health Organization (WHO) recommends that women with HIV receive cervical cancer screening with HPV testing at least every 3 years if resources are available; for countries with fewer resources, visual inspection with acetic acid (VIA) is an acceptable screening alternative.^8^ Screening with cervical cytology (ie, Pap testing) is only recommended for countries that have already achieved high cytology coverage and quality indicators. Recent guidelines from the American Society for Clinical Oncology recommend HPV testing at the time of HIV diagnosis and subsequently at an interval of every 2–3 years if resources are available; for lower-resource settings, screening for HIV-infected women is recommended twice as often as in the general population.^9^ In South Africa—where screening guidelines recommend cytology-based screening, with phasing in of HPV testing based on resource availability—the principles of equity, quality, efficiency, and sustainability have been adopted by the Department of Health.^10^ Yet data on the cost-effectiveness of different screening and management strategies in HIV-infected women are limited.

To inform ongoing discussions about clinical guidelines, optimal resource use, and the integration of women's health interventions with HIV-related care in South Africa and other low-resource settings with a high burden of HIV, we evaluated the cost-effectiveness of different Pap and HPV screening and management algorithms among HIV-infected women in South Africa.

## METHODS

### Epidemiologic Modeling

**FIGURE 1.**
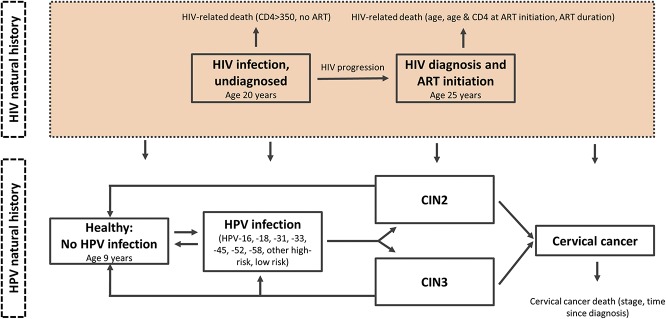
Model schematic for the natural history model of HPV infection and progression to cervical cancer in the presence of HIV infection. Individual girls enter the model at the age of 9 years, before HPV and HIV infection. Each month, they face probabilities of transitioning between mutually exclusive HPV-related health states, including type-specific HPV infection (HPV types 16, 18, 31, 33, 45, 52, 58, other oncogenic types, and low-risk types), CIN grades 2 or 3 (CIN2, CIN3), and cervical cancer (local, regional, and distant stages). Each month, death can occur from noncervical causes or from cervical cancer after its onset (depending on stage and time since diagnosis). Transitions between health states may vary by duration of infection or CIN, HPV type, age, history of previous HPV infection, and patterns of screening. Women are infected with HIV at the age of 20 years. On HIV infection, women face excess mortality rates based on CD4^+^ cell count and rate of HIV progression.^14^ After HIV presentation and diagnosis at the age of 25 years (CD4^+^ cell count 350 cells/µL), women begin ART immediately and face excess mortality from HIV based on age, age at ART initiation, CD4^+^ category at ART initiation, and duration on ART.^7^

We modified an existing individual-based microsimulation model of HPV infection and cervical cancer^11,12^ to reflect the burden of HPV in HIV-infected women in South Africa (Fig. 1) and used the model to project the health and economic outcomes associated with different Pap and HPV-based screening and management algorithms. Individual girls enter the model at the age of 9 years, before HPV and HIV infection. Each month, they face probabilities of transitioning between mutually exclusive HPV-related health states, including type-specific HPV infection, cervical intraepithelial neoplasia grades 2 or 3 (CIN2, CIN3), and cervical cancer (local, regional, and distant stages). Transitions between health states may vary by duration of infection or CIN, HPV type, age, history of previous HPV infection, and patterns of screening. The model keeps track of each individual woman's health status and resource use over time and then aggregates cost and health outcomes at the population level.

Because of limited data on the interaction between HPV and HIV, we assumed a similar course of HIV infection and progression for all women in the model (see Table 2, Supplemental Digital Content, http://links.lww.com/QAI/B185). Women in the model are infected with HIV at the age of 20 years, approximating the peak HIV incidence in South Africa.^13^ Based on HIV progression estimates in the absence of ART^14,15^ and estimates of CD4^+^ cell count at HIV presentation,^16^ we optimistically assumed (as HIV testing and ART access increase) that women would be diagnosed with HIV around the age of 25 years, at a CD4^+^ cell count of approximately 350 cells/µL. Before HIV acquisition at the age of 20 years, background mortality rates for uninfected women were drawn from the THEMBISA model (version 2.5).^13^ On HIV infection at the age of 20 years, women face excess mortality rates dependent on CD4^+^ cell count and rate of HIV progression.^14^ On HIV detection at the age of 25 years, women are assumed to begin ART immediately and then face excess mortality from HIV based on a relative survival model that considers current age, age at ART initiation, CD4^+^ category at ART initiation, and duration on ART.^7^ Excess mortality due to cervical cancer was derived from estimates of 5-year survival in middle-income countries with a low burden of HIV^17^ multiplied by hazard ratios from cervical cancer mortality in HIV-infected versus uninfected women.^18^ Time-dependent survival by stage (for 1-, 2-, and 3- to 10-year survival) was derived from cervical cancer survival estimates in HIV-infected women in Botswana.^19^ After 10 years following cancer treatment, we assumed women no longer faced excess mortality due to cervical cancer.

**FIGURE 2.**
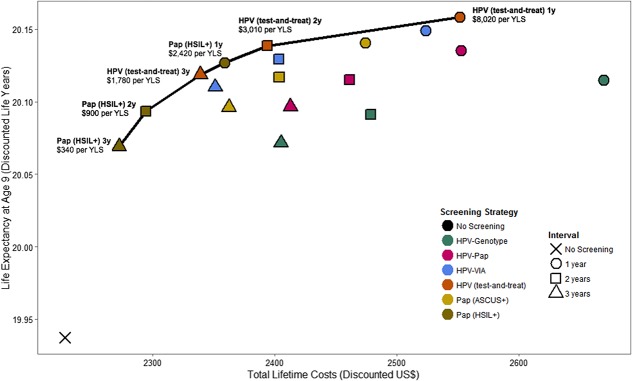
Cost-effectiveness analysis: base case results. The graph displays the discounted lifetime costs (*x*-axis; in 2017 US$) and life expectancy (*y*-axis) associated with each screening strategy delivered at intervals of every 1 (1y, circles), 2 (2y, squares), or 3 (3y, triangles) years. Screening strategies included Pap (HSIL+) (Pap testing at a referral threshold of atypical squamous cells cannot rule out high-grade/high-grade squamous intraepithelial lesions or worse); Pap (ASCUS+) (Pap testing at a referral threshold of atypical squamous cells of undetermined significance or worse); HPV (test-and-treat) (HPV testing followed by treatment for all HPV-positive women); HPV-VIA (HPV testing followed by VIA for HPV-positive women, and treatment for all HPV-positive/VIA-positive women); HPV-Pap (HPV testing followed by Pap triage of HPV-positive women, and treatment for all HPV-positive/ASCUS+ women); and HPV genotyping (HPV testing followed by genotyping for HPV-positive women, with HPV16/18-positive women referred to treatment and other oncogenic types referred to colposcopy). The cost-effectiveness associated with a change from one strategy to a more costly alternative is represented by the difference in cost divided by the difference in life expectancy associated with the 2 strategies. The curve indicates the strategies that are efficient because they are more effective and either (1) cost less or (2) have a more attractive cost-effectiveness ratio than less effective options. The ICER is the reciprocal of the slope of the line connecting the 2 strategies under comparison.

To inform transitions between HPV-related health states, baseline “prior” transition probabilities were estimated from large epidemiologic studies in predominantly HIV-uninfected populations, as previously documented.^11,12^ Our modeling approach assumes similarities in the natural history of HPV regardless of setting but allows for differences in HPV incidence (due to sexual behavior) and type-specific immunity. To reflect the greater risk of HPV infection and progression (to CIN and cancer) and the decreased likelihood of HPV clearance and CIN regression in HIV-infected women, as well as parameter uncertainty, we modified the previously described natural history model by setting plausible ranges for factors to apply to baseline transition probabilities derived from longitudinal studies, guided by previous work modeling HPV infection in the general population of women in several low- and middle-income countries with a low burden of HIV^12,20^ and published hazard and risk ratios in HIV-infected versus HIV-uninfected women.^21^ The model underwent repeated model simulations in the absence of any intervention, and for each simulation, single random values from a uniform distribution spanning the plausible range for each factor were selected and applied to the relevant baseline probability to create a unique natural history input parameter set. We then computed a goodness-of-fit score by summing the log likelihood of model-projected outcomes for each unique parameter set to represent the quality of fit to epidemiologic data (ie, the calibration targets) on age-specific prevalence of oncogenic HPV and the proportion of HPV type-specific infections in CIN3 and cervical cancer among women with HIV in South Africa.^22–24^ We selected the 50 top-fitting parameter sets for analyses and calculated the expected value, as well as the range of values, for all outcomes. Model fit to calibration targets is displayed in Figures 1 and 2, Supplemental Digital Content, http://links.lww.com/QAI/B185. Data sources used to inform the factor search space and the ranges for each transition probability value are provided in Table 1, Supplemental Digital Content, http://links.lww.com/QAI/B185. Model validations to CIN2/3 prevalence and cervical cancer incidence in South Africa are presented in Figures 3 and 4, Supplemental Digital Content, http://links.lww.com/QAI/B185.

### Screening and Management Algorithms

Screening and management algorithms were based on recently drafted guidelines for South Africa^10^ and WHO guidelines for HIV-infected women.^8^ Strategies included (1) liquid-based cytology with referral to colposcopy for atypical squamous cells of undetermined significance or worse (ASCUS+) [“Pap (ASCUS+)”]; (2) liquid-based cytology with referral to colposcopy for atypical squamous cells cannot rule out high-grade/high-grade squamous intraepithelial lesions or worse (HSIL+) [“Pap (HSIL+)”]; (3) HPV DNA testing with referral to treatment for all HPV-positive women (“HPV test-and-treat”); (4) HPV DNA testing with triage to VIA for all HPV-positive women, and treatment for all women who are both HPV-positive and VIA-positive (“HPV-VIA”); (5) HPV DNA testing with triage to Pap for all HPV-positive women, and referral to colposcopy for all women who are both HPV-positive and ASCUS+ (“HPV-Pap”); and (6) HPV DNA testing followed by HPV16/18 genotyping for HPV-positive women, with referral to treatment for 16/18-positive women and referral to colposcopy for women with other oncogenic types (“HPV16/18 genotyping”). Screening coverage was 70% of the target population. Of those screened, 85% were assumed to receive the strategy of interest (strategies 1 through 6, above), whereas 15% were assumed to receive a separate VIA strategy, to represent the potential availability of VIA followed by treatment for women without access to Pap or HPV-based strategies; we weighted cost and health outcomes accordingly. Screening and management algorithms are displayed in Figures 5–11, Supplemental Digital Content, http://links.lww.com/QAI/B185. All strategies were considered at 1-, 2-, and 3-year intervals.

For HPV-testing strategies, we assumed provider-collection of samples. For HPV testing followed by either visual or Pap triage, women who were HPV-positive but triage test–negative were assumed to receive repeat HPV testing in 1 year. After colposcopy, women with normal or CIN grade 1 (CIN1) on histology were referred to repeat screening in 1 year; those with CIN2 or higher were referred to treatment. Treatment included cryotherapy for eligible women, and large loop excision of the transformation zone (LLETZ) for women who were determined to be ineligible for cryotherapy. Screening test performance and treatment effectiveness values were based on studies in HIV-infected women.^22,25–33^ Compliance with each recommended clinical encounter was 85% relative to the previous visit. Values for screening and treatment variables are displayed in Table 1.

**TABLE 1.**
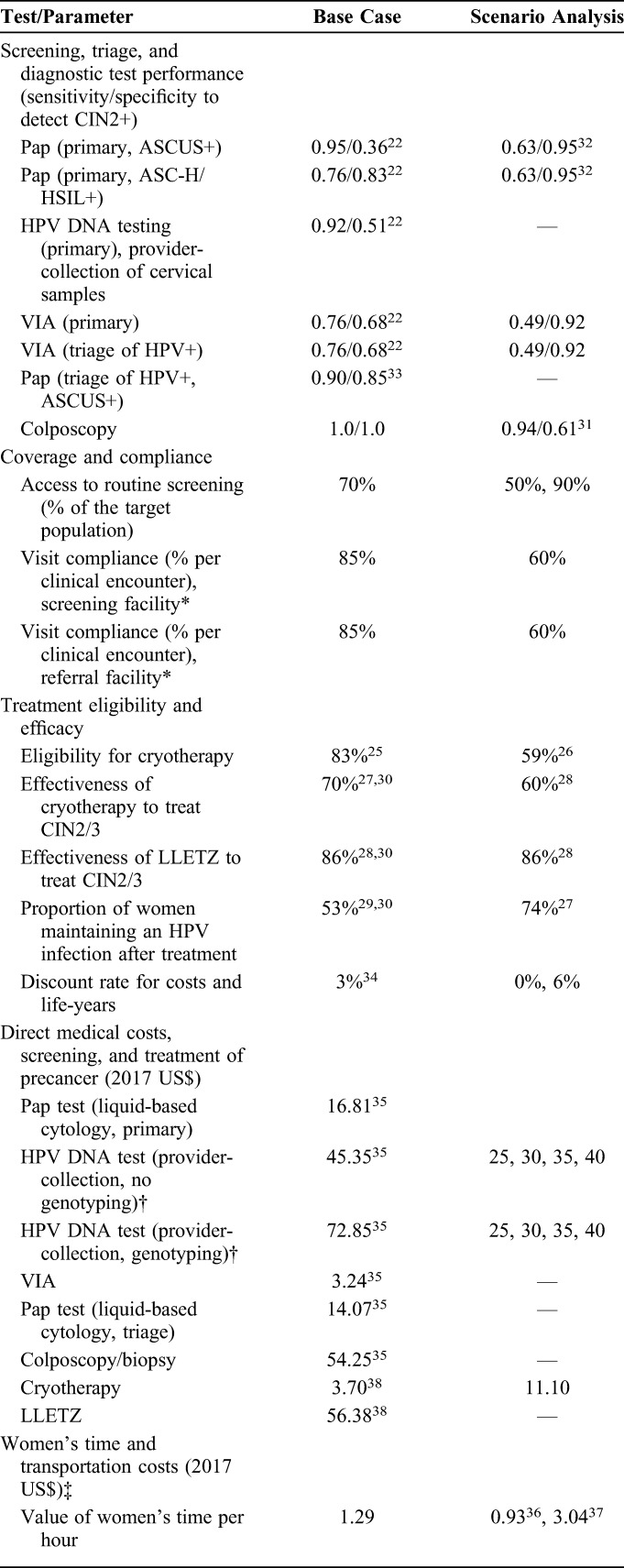

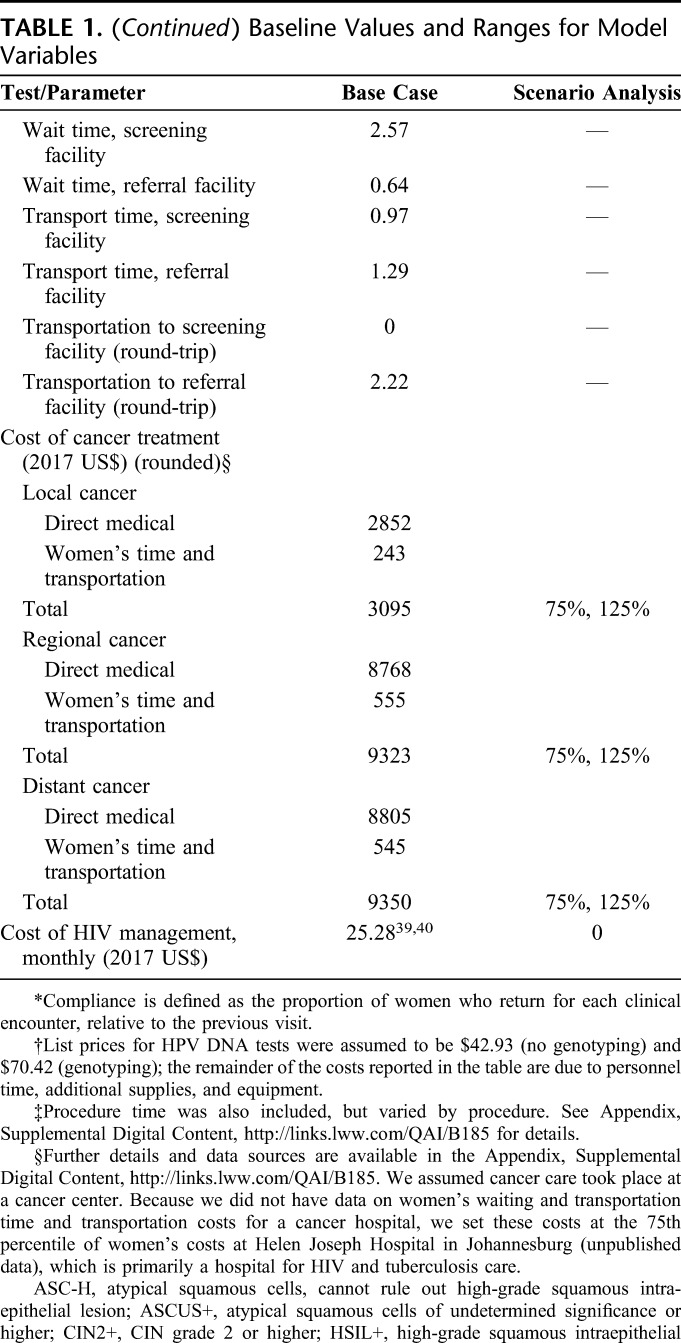
Baseline Values and Ranges for Model Variables

### Estimation of Costs and Cost-Effectiveness

Consistent with guidelines for cost-effectiveness analysis, we adopted a modified societal perspective^34^; costs were estimated from in-country data sources and included direct medical costs (personnel time, consumable supplies, equipment, and South Africa National Health Laboratory Service/National Department of Health service charges to estimate laboratory costs), direct nonmedical costs (women's transportation), and women's time (Table 1).^35–38^ Cost data were collected in local currency units and converted to 2017 US dollars (US$) using consumer price indexes and average annual official exchange rates to report findings for global policy makers. Because HPV DNA and HPV genotyping tests have not yet been procured by the government of South Africa, list prices (approximately $43 and $70, respectively) did not necessarily reflect economies of scale from bulk purchasing. Each month, beginning at HIV diagnosis, women incurred the average monthly outpatient cost per adult on ART and average monthly inpatient cost for individuals on ART with a CD4 count above 350 cells/µL.^39,40^ Further details and data sources are provided in the Supplemental Digital Content, http://links.lww.com/QAI/B185.

Model outcomes included the absolute lifetime risk of cervical cancer incidence and mortality, life expectancy, and lifetime costs; costs and life-years were discounted at an annual rate of 3%.^34^ We then calculated incremental cost-effectiveness ratios (ICERs). An ICER is the additional cost of a strategy divided by its additional health benefit, compared with the next most costly strategy after eliminating strategies that are dominated (ie, either more costly and less effective, or having higher ICERs than more effective strategies). Across the 50 top-fitting input parameter sets, the ICER was calculated as the ratio of the mean cost divided by the mean health effect. We considered the following possible cost-effectiveness thresholds as a benchmark to indicate strategies that provide good value for money: (1) South Africa's per capita gross domestic product (GDP) (2016 US$5270)^41^; (2) 50% of South Africa's per capita GDP (2016 US$2640); and (3) the cost per disability-adjusted life-year averted for extending ART eligibility from adult patients with CD4 counts of 350 cells/µL or less to patients with CD4 counts of 500 cells/µL or less—an approximation of the opportunity cost of HIV care in South Africa—as estimated by a recent cost-effectiveness analysis using 12 mathematical models (high estimate for South African models: 2016 US$1190).^42^

Scenario analyses assessed the impact of alternate parameter values and assumptions on cost-effectiveness results (Table 1).

## RESULTS

The impact on cervical cancer incidence and mortality, lifetime cost, life expectancy, and ICER for each screening strategy is presented in Table 2. Beginning at HIV diagnosis at the age of 25 years, HPV test-and-treat at 1-year intervals was the most effective strategy (ie, yielded the greatest reductions in cervical cancer incidence and mortality; greatest years of life saved), reducing the absolute lifetime risk of cervical cancer by 64% compared with no screening. HPV-VIA was nearly as effective, reducing cancer risk by 61%. As screening intervals expanded from 1 to 3 years, the impact on cancer incidence was reduced; HPV test-and-treat every 3 years reduced cancer risk by 49.1%, whereas HPV-VIA every 3 years reduced cancer risk by 46.8%. For any given screening interval, HPV test-and-treat was the most effective strategy followed by HPV-VIA, whereas Pap (HSIL+) was the least effective strategy due to low test sensitivity and the high number of required visits for screening and management. HPV-Pap was more effective at reducing cancer risk than Pap (ASCUS+) or HPV16/18 genotyping at the same interval, except at 1-year interval when Pap (ASCUS+) was slightly more effective.

**TABLE 2.**
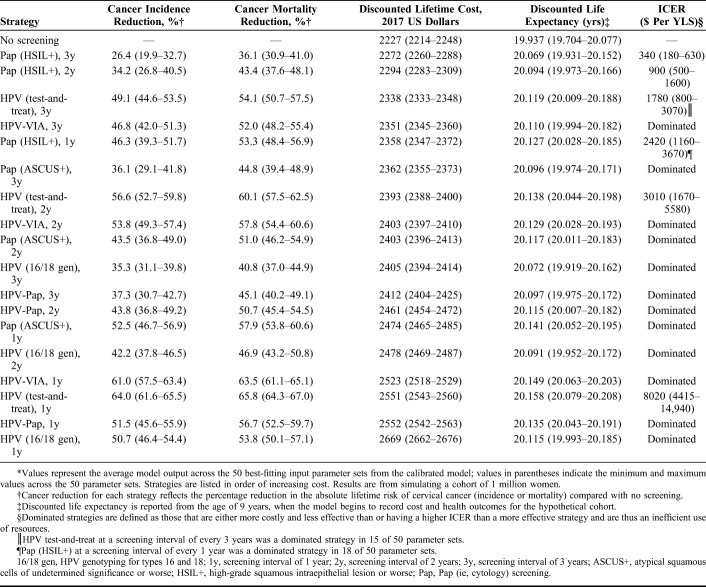
Cervical Cancer Impact, Costs, and ICERs of Screening in HIV-Infected Women*

Pap (HSIL+) every 3 years had the lowest ICER [$340 per year of life saved (YLS)], followed by Pap (HSIL+) every 2 years ($900 per YLS), but these strategies also were the least effective. More effective strategies that were also efficient (ie, nondominated strategies that lie on the efficiency frontier) included HPV test-and-treat every 3 years ($1780 per YLS), Pap (HSIL+) every year ($2420 per YLS), HPV test-and-treat every 2 years ($3010 per YLS), and HPV test-and-treat every year ($8020 per YLS) (Fig. 2). The optimal strategy—the most effective strategy with an ICER below a specified cost-effectiveness threshold—varied according to threshold (Table 3). At a threshold of $1190, Pap (HSIL+) every 2 years was optimal, reducing cancer incidence by 34.2%. At a higher threshold of $2640, Pap (HSIL+) every year was optimal, reducing cancer incidence by 46.3%. Given the threshold of per capita GDP ($5270), HPV test-and-treat every 2 years was the optimal strategy, reducing cancer incidence by 56.6%.

**TABLE 3.**
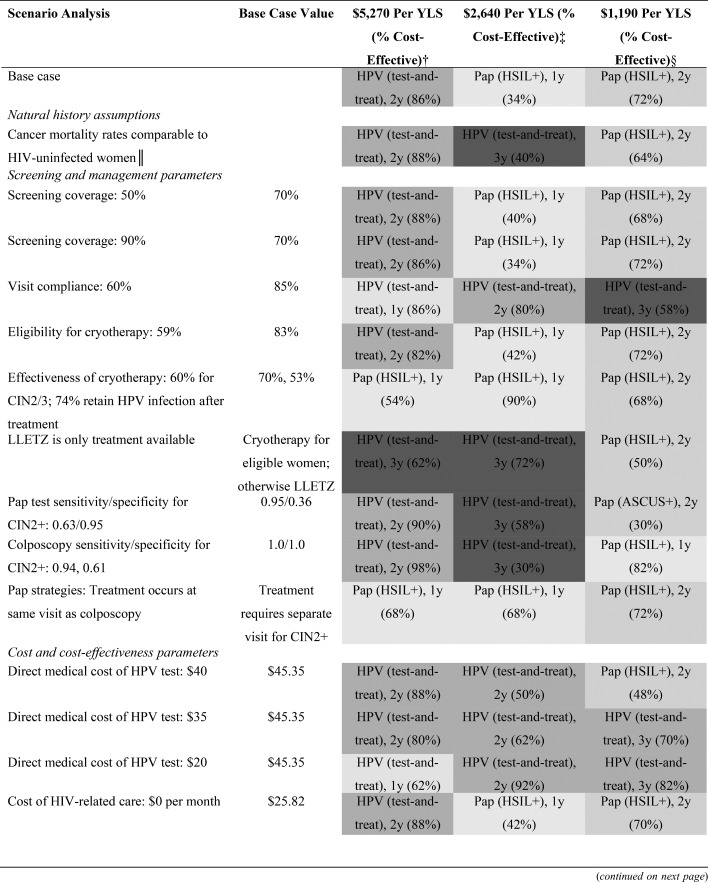

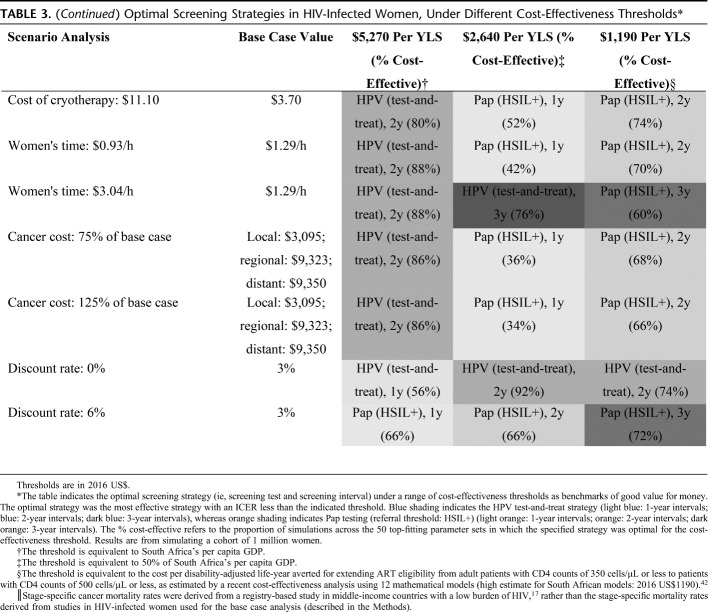
Optimal Screening Strategies in HIV-Infected Women, Under Different Cost-Effectiveness Thresholds*

Results from scenario analyses are presented in Table 3. The optimal strategy according to cost-effectiveness threshold did not change when we varied assumptions around screening coverage, reduced eligibility for cryotherapy, reduced cost of HIV-related care, increased cost of cryotherapy, reduced women's time costs, or reduced or increased cancer costs. HPV-based screening strategies became optimal at a lower threshold when we assumed reduced cancer mortality rates, reduced visit compliance, reduced diagnostic performance of colposcopy, reduced Pap test performance, increased women's time cost, reduced HPV test cost, assumed LLETZ was the only available treatment, or assumed a discount rate of 0%. Pap-based screening (primarily at an HSIL+ referral threshold) displaced HPV testing as the optimal strategy below the per capita GDP threshold when effectiveness of cryotherapy decreased, when colposcopy and treatment were assumed to always occur at the same visit, or when the discount rate was 6%.

## DISCUSSION

This model-based analysis found that, for women presenting with HIV at the age of 25 years and receiving prompt access to ART in South Africa, HPV testing every 2 years followed by treatment for HPV-positive women was very cost-effective according to a cost-effectiveness threshold based on per capita GDP, reducing cervical cancer risk by 56.6%. At lower willingness-to-pay thresholds (including 50% of per capita GDP and $1190), Pap testing (HSIL+ threshold) every 1–2 years would be considered optimal but would yield much lower reductions in cervical cancer risk (46.3% and 34.2%, respectively). Optimal strategies under various cost-effectiveness benchmarks were robust across many scenario analyses. However, when the high direct medical cost of the HPV test in the base case was reduced to $35, HPV test-and-treat at 2- or 3-year intervals was the optimal strategy for all benchmarks considered. Furthermore, because of the lower number of required visits for this strategy compared with Pap-based strategies, less favorable assumptions regarding visit compliance improved the relative attractiveness of HPV test-and-treat.

To determine whether an intervention should be included in a package of services, a benchmark of health opportunity costs, or cost-effectiveness threshold, must be established. The WHO Commission on Macroeconomics and Health suggests that interventions with ICERs less than per capita GDP are “very cost-effective” and less than 3 times per capita GDP are “cost-effective.^41^” Yet recent analyses suggest that accepting interventions according to per capita GDP may displace interventions yielding substantial health benefits with less effective interventions.^43^ In the absence of a formal threshold based on empirical data from South Africa, we considered alternative thresholds, including 50% of per capita GDP and $1190, which represents the ICER (from a recent modeling study in South Africa) of expanding ART coverage from all adults with CD4 counts below 350 cells/µL to those with CD4 counts below 500 cells/µL. In addition to consideration of value for money, it is critical to consider health equity in determining what services should be available.

In South Africa, access to cryotherapy is limited, but recently drafted guidelines rely heavily on this form of treatment, and our analysis assumed that most eligible women would receive cryotherapy.^10^ The effectiveness of treatment in HIV-infected women is uncertain. One recent randomized trial found that HIV-infected women with CIN2/3 were more likely to experience recurrence after cryotherapy than after LEEP at 12 and 24 months after treatment, as measured by HSIL+ on cytology; another trial in South Africa found that more women had recurrent CIN2+ after cryotherapy than LEEP at 6 months, but by 12 months, the difference was not significant.^30,44^ When we assumed all women were treated with LLETZ instead of cryotherapy, HPV test-and-treat every 3 years was optimal at cost-effectiveness thresholds of per capita GDP and 50% of per capita GDP; less frequent screening was needed because of the assumed greater effectiveness of treatment. Although our analysis included the costs associated with potential overtreatment of screen-positive women in HPV test-and-treat strategies, we did not consider the impact of potential adverse reproductive outcomes. Guidelines will need to weigh safety, effectiveness, and logistical considerations (such as cost, provider training, and capacity) in recommending surgical versus ablative treatments for women with HIV.

Because of the complexities of modeling HIV and HPV coinfection, few studies have evaluated the cost-effectiveness of cervical cancer screening in HIV-infected women in a low- or middle-income country. Vanni et al^45^ found that HPV testing every 1–2 years followed by Pap triage of HPV-positive women was cost-effective in Brazil but did not consider HPV testing with treatment for all HPV-positive women. Lince-Deroche et al^35^ estimated the cost per case of CIN2+ detected associated with screening in South Africa and found that HPV testing followed by colposcopy of HPV-positive women had the highest cost per case detected, whereas VIA had the lowest; however, this analysis did not consider the cost offsets of prevented cancers.

There are several limitations to this analysis. We did not have data to inform HPV transitions stratified by CD4 count, viral load, or ART status and thus assumed a common trajectory of HIV disease in the modeled cohort. Further research on the time-dependent impact of ART on HPV and cervical cancer transitions will inform future modeling efforts. Furthermore, our calibration to oncogenic HPV prevalence relied on data from the VICAR study of HIV-infected women in Johannesburg, and we thus could not calibrate to the prevalence of HPV for women younger than 20 years before HIV infection, nor can we assume generalizability to HIV-infected women in other parts of the country. However, by achieving good fit to HPV prevalence beginning at the age of 20 years, when screening does not begin until the age of 25 years, we believe the model reflects appropriate clearance versus persistence of earlier HPV infections. Furthermore, our validation exercises examining model-projected cervical cancer incidence relative to registry data for the general population are consistent with hazard ratios gleaned from studies in HIV-infected versus uninfected women.^46^ Although we modeled screening strategies based on WHO recommendations and recently drafted guidelines in South Africa, HPV testing is not widely available in South Africa at this time. Although there are plans for scale-up as resources become available, the delivery of HPV testing and management may need to be refined based on system resources or improved for more efficient delivery. We did not consider reduced screening intervals after a specific number of negative screening tests, nor did we model switching from Pap-based to HPV-based screening or cotesting after the age of 30 years, as US guidelines suggest.^47^ We did not consider the future impact of HPV vaccination of girls, which was introduced in South Africa in 2014.

As women live longer due to improved access to life-saving ART, they will likely continue to face an elevated risk of cervical cancer. In South Africa, expanded access to organized cervical cancer screening with cytology has reduced cervical cancer incidence in HIV-infected women^4^; however, tremendous disparities in access to screening remain. Our model-based analysis demonstrates that cervical cancer screening is likely to be both effective and very cost-effective in HIV-infected women in South Africa. If sufficient resources are available, HPV testing at 2-year intervals can achieve greater reductions in cancer risk than Pap-based strategies in South Africa through increased detection of precancer and fewer visits per screening episode. As the demand for HPV testing rises and test costs decline, a shift toward HPV-based test-and-treat strategies in South Africa and other low-resource settings with a high burden of HIV may improve access and save lives. Health services research will be necessary to integrate cervical cancer prevention with HIV-related services through community outreach, task-shifting to nonphysicians, and strengthening information systems to track patients^48^; integrating and improving service delivery for high-risk women is a tremendous opportunity to transform the health care system and reduce health disparities. We present optimal strategies under a range of benchmarks for cost-effectiveness to stimulate action from international donors, advance policy discussions, and inform implementation efforts—so that women who are saved from HIV will not die of cervical cancer.

## Supplementary Material

SUPPLEMENTARY MATERIAL
