# Supplementary material for: Cost-Effectiveness of Cervical Cancer Screening in Women Living With HIV in South Africa: A Mathematical Modeling Study
Source: J Acquir Immune Defic Syndr. 2018 Jun 15;79(2):195–205. doi: 10.1097/QAI.0000000000001778 (PMC6143200; doi:10.1097/QAI.0000000000001778)
Supplement: SUPPLEMENTARY MATERIAL [file qai-79-195-s001.docx]

**Supplementary Appendix:**

**Cost-effectiveness of cervical cancer screening in women living with HIV in South Africa**

**Natural history model**

Derivation of baseline model input parameters for the general HPV natural history model have been described in detail elsewhere; these are based on longitudinal data.[^1^](#_ENREF_1)^,^[^2^](#_ENREF_2) Ranges for factors, by which baseline values are multiplied to derive transition probabilities for the calibrated natural history of HPV infection and cervical carcinogenesis in South African women infected with HIV at age 20 years, from the 50 top-fitting parameter sets are displayed in Supplementary Table 1.

**Model calibration**

Model fit to the epidemiologic data (i.e., calibration targets) on age-specific oncogenic HPV prevalence, and HPV type distribution in CIN3 and invasive cervical cancer in HIV-infected women in South Africa, is displayed in Supplementary Figures 1 and 2, respectively.

**Model validation**

Following calibration, we performed validation exercises to compare the natural history model outputs to expected outcomes from the literature. Findings for the age-specific prevalence of CIN2/3 in HIV-infected women in South Africa are displayed in Supplementary Figure 3. Findings for age-specific cervical cancer incidence are displayed in Supplementary Figure 4.

We found that the median duration of an HPV16 infection in the model was 23.6 months. A study of self-collected swabs in Cape Town found that, among HIV-infected women, 38% retained an HPV16/18 infection at 12 months following detection.^[3](#_ENREF_3)^ The model’s longer median duration of HPV likely reflects the lack of censoring in the model (as opposed to the study) and the fact that HPV16 and 18 infections in the study were pooled, and may not reflect the greater persistence of HPV16 relative to HPV18. The median duration of HPV18 infections in the model was 13.4 months. A study by Ahdieh and colleagues found that 36% of HPV16 and 29% of HPV18 infections persisted in HIV-infected women with CD4+ counts ≥200 cells/ul who were followed for a median of 5 semi-annual visits (~2.5 years); among women with CD4+ counts <200, 56% of HVP16 infections and 100% of HPV18 infections persisted over the same period.^[4](#_ENREF_4)^

A study of HIV-infected cervical cancer cases in South Africa, Ghana, and Nigeria noted 4%, 78%, and 18% of patients presented with local, regional, and distant cancer, respectively.^[5](#_ENREF_5)^ A study in Botswana found that among HIV-infected women with cervical cancer, approximately 13%, 77%, and 10% presented with local, regional, and distant cancer, respectively ^[6](#_ENREF_6)^. Model outputs projected that 19%, 72%, and 9% of women present with local, regional, and distant cancer in the absence of screening.

**Cost data**

Cost data were collected in local currency units and converted to 2017 U.S. dollars (US$) using consumer price indexes and average annual official exchange rates.[^7^](#_ENREF_7) Costs were estimated from in-country data sources and included direct medical costs (personnel time, consumable supplies, equipment, and South Africa National Health Laboratory Service/National Department of Health service charges to estimate laboratory costs),[^8^](#_ENREF_8)^,^[^9^](#_ENREF_9) direct non-medical costs (women’s transportation), and women’s time.

Women’s costs for screening, diagnostic follow-up, and treatment of precancer were based on a survey of HIV-infected women attending HIV care clinics at Helen Joseph hospital (a tertiary center in Johannesburg) and HIV-infected and HIV-uninfected women who attended HIV care clinics or received sexual and reproductive health services at primary health clinics in the community. Women who reported a Pap test in the past year were interviewed to ascertain time and costs (Helen Joseph hospital: n=87; community clinics: n=36).[^10^](#_ENREF_10) Interviews were conducted in 2016.

*Women’s transportation*

Median round-trip transportation to the primary health clinic cost 2017 US$0, as women tended to walk (interquartile range: $0.00-$1.17). Median round-trip transportation to Helen Joseph hospital cost US$2.22 (interquartile range: $1.27-$3.49). In the cost-effectiveness analysis, we considered the median primary health clinic transportation cost for all screening visits, and the median Helen Joseph hospital transportation cost for all diagnostic and treatment visits.

*Women’s time*

Women’s time spent traveling and waiting for care at a primary health clinic or Helen Joseph hospital was drawn from the same survey data. The median time spent traveling (round-trip) was 45 minutes (interquartile range: 20 minutes-60 minutes) for primary health clinics and 60 minutes (interquartile range: 40 minutes-120 minutes) for Helen Joseph hospital. The median time spent waiting at the clinics was 120 minutes (interquartile range: 60 minutes-157.5 minutes) and at the hospital was 30 minutes (interquartile range: 30 minutes-60 minutes). In the cost-effectiveness analysis, we considered the median travel and wait times at the clinics (for screening) and at Helen Joseph hospital (for diagnostic and treatment visits).

Women’s time costs for receiving care (depending upon procedure) were drawn from a published study of screening costs in HIV-infected women in Johannesburg[^8^](#_ENREF_8) and a study of treatment costs in Johannesburg.[^9^](#_ENREF_9) We assumed women spent the following times according to procedure: liquid-based cytology, 28 minutes; HPV DNA testing, 24 minutes; VIA: 12 minutes; receiving screening results: 8.33 minutes; cryotherapy: 40 minutes; LLETZ: 25 minutes; colposcopy with biopsy: 21 minutes.

Women’s time was valued based on the survey of women attending primary health clinics or Helen Joseph hospital. The survey elicited information on lost income for attending screening at either type of facility, and the median lost income for all patients was assumed to be equivalent to one day’s wages. We thus inferred an hourly wage of US$1.29. We varied this in sensitivity analyses using a lower bound of $0.93 per hour (from minimum wage data reported by the South African National Department of Labor)[^11^](#_ENREF_11) and an upper bound of US$3.04 per hour (from International Labor Organization estimates of average monthly earnings for female employees) [^12^](#_ENREF_12).

*HIV care*

The costs of HIV treatment were based on the average outpatient cost per adult on ART from the National ART Cost Model of South Africa.[^13^](#_ENREF_13) Average inpatient cost per patient year for individuals on ART with a CD4 count above 350 cells/µl was derived from a study in rural and urban settings in South Africa, and was apportioned each month.[^14^](#_ENREF_14)

*Cancer treatment*

The costs of delivering the standard of care for cervical cancer staging and treatment is presented in in Supplementary Table 3 and was based on fee schedules and price lists in South Africa, as well as expert opinion. In the model, cervical cancer may present at local, regional, or distant stages. All women were assumed to incur staging costs. We assumed, based on estimates of the proportion of women who present at each FIGO stage in South Africa, that for local cancers, 26.7% of women incur the costs of Stage 1a1, 4.8% incur the costs of 1a2, 48.1% incur the costs of 1b1, and 20.4% incur the costs of 1b2. For regional cancers, we assumed that 1.4% incur the costs of 2a, 35.4% incur the costs of 2b, and 63.2% incur the costs of 3b. Of women presenting with distant cancers, 44.5% incurred the cost of 4a and 55.5% incurred the costs of 4b.

For women’s time and transportation costs incurred for cancer treatment, we used the 75^th^ percentile of transportation costs and travel time from the survey of HIV-infected women receiving care at Helen Joseph hospital, or US$3.49 and 120 minutes, respectively, for each round-trip visit to the cancer center. For staging, we assumed 4 visits, each of the same duration as a visit for colposcopy with biopsy (21 minutes). For surgical treatment (FIGO stages 1a1, 1a2, 1b1, 2a), we assumed 3 inpatient days (8 hours each) for simple hysterectomy and 7 inpatient days for radical hysterectomy. Each surgical procedure was followed by a 6-week post-op follow-up visit, a 6- and 12-month follow-up visit including Pap tests, and results visits for the two Pap tests. For radiation and chemotherapy (FIGO stages 1b2, 2a, 2b, 3a, 3b, 4a, 4b) we assumed 31 outpatient visits requiring the full 8-hour day for traveling, waiting for, and receiving care, plus an additional 4 follow-up visits.

**Supplementary Table 1. Transition probabilities in the natural history model of HPV infection in women infected with HIV at age 20 years.**

| **Progression of Healthy to HPV Infection** [**^15-22^**](#_ENREF_15) | | | | |
| --- | --- | --- | --- | --- |
| **HPV Type, by Age (Years)** | Baseline Value | | Range of Factor Values Among Top 50 Parameter Sets | |
|  | Min^a^ | Max^a^ | Min | Max |
| **HPV 16** | | | | |
| <21 | 0 | 0.00186 | 5.08182 | 17.9236 |
| 21-24 | 0.000903 | 0.00125 | 5.62139 | 17.8136 |
| 25-29 | 0.000782 | 0.00087 | 5.0528 | 14.207 |
| 30-49 | 0.000602 | 0.00078 | 6.09803 | 15.9277 |
| >= 50 | 0.000217 | 0.00059 | 5.56754 | 15.5884 |
|  |  |  |  |  |
|  |  |  |  |  |
| **HPV 18** | | | | |
| <21 | 0 | 0.00116 | 5.29753 | 21.2746 |
| 21-24 | 0.0011 | 0.001173 | 5.58122 | 21.9604 |
| 25-29 | 0.0006 | 0.001 | 5.02746 | 21.6739 |
| 30-49 | 0.000301 | 0.00058 | 5.05024 | 19.9533 |
| >= 50 | 0.000109 | 0.000295 | 5.92947 | 19.7387 |
|  |  |  |  |  |
| **HPV 31** | | | | |
| <21 | 0 | 0.001428 | 5.03592 | 17.435 |
| 21-24 | 0.0009 | 0.001364 | 5.01263 | 17.7383 |
| 25-29 | 0.0006 | 0.000805 | 5.00125 | 16.2025 |
| 30-49 | 0.000301 | 0.00055 | 5.29898 | 15.8345 |
| >= 50 | 0.000109 | 0.000295 | 5.04411 | 15.886 |
|  |  |  |  |  |
| **HPV 33** | | | | |
| <21 | 0.000786 | 0.000786 | 5.20122 | 15.7543 |
| 21-24 | 0.00069 | 0.00069 | 5.66214 | 15.9463 |
| 25-29 | 0.00036 | 0.00036 | 5.06978 | 11.8541 |
| 30-49 | 0.00022 | 0.00022 | 5.06609 | 11.9436 |
| >= 50 | 0.000128 | 0.000128 | 5.04116 | 11.6927 |
|  |  |  |  |  |
| **HPV 45** | | | | |
| <21 | 0 | 0.001 | 5.11585 | 17.9857 |
| 21-24 | 0.00041 | 0.0008 | 5.3889 | 17.5586 |
| 25-29 | 0.000315 | 0.00037 | 5.0709 | 14.8285 |
| 30-49 | 0.000161 | 0.000305 | 5.10515 | 15.8288 |
| >= 50 | 0.000054 | 0.00015 | 5.08241 | 15.9169 |
|  |  |  |  |  |
| **HPV 52** | | | | |
| <21 | 0 | 0.001186 | 5.16536 | 13.8596 |
| 21-24 | 0.00027 | 0.0008 | 5.12337 | 13.8957 |
| 25-29 | 0.000184 | 0.000254 | 5.07651 | 11.884 |
| 30-49 | 0.00015 | 0.000173 | 5.09463 | 11.8316 |
| >= 50 | 0.000054 | 0.000148 | 5.26916 | 11.7788 |
|  |  |  |  |  |
| **HPV 58** | | | | |
| <21 | 0 | 0.001189 | 5.11934 | 17.3279 |
| 21-24 | 0.00059 | 0.0011 | 5.20651 | 17.6771 |
| 25-29 | 0.000465 | 0.00055 | 5.04253 | 13.6454 |
| 30-49 | 0.000244 | 0.00045 | 5.30659 | 15.9611 |
| >= 50 | 0.000054 | 0.000239 | 5.20997 | 15.995 |
|  |  |  | 5.29753 | 21.2746 |
| **Other Oncogenic Types** | | | | |
| <21 | 0 | 0.00247 | 5.01126 | 13.7189 |
| 21-24 | 0.001804 | 0.0023 | 5.03927 | 13.9381 |
| 25-29 | 0.0012 | 0.0017 | 5.01532 | 13.1104 |
| 30-49 | 0.000602 | 0.0011 | 5.25958 | 11.9726 |
| >= 50 | 0.000217 | 0.00059 | 5.04666 | 11.8949 |
|  |  |  |  |  |
| **Nononcogenic Types** | | | | |
| <21 | 0 | 0.00261 | 5.41376 | 14.9171 |
| 21-24 | 0.00255 | 0.00262 | 5.09345 | 14.4265 |
| 25-29 | 0.002 | 0.0025 | 5.02335 | 11.8837 |
| 30-49 | 0.00085 | 0.00186 | 5.19679 | 14.6889 |
| >= 50 | 0.000217 | 0.0008 | 5.0369 | 14.891 |

| **HPV Progression to CIN2** [**^23^**](#_ENREF_23) | | | |
| --- | --- | --- | --- |
| **HPV Type and Time Since Infection (Months)** | Baseline Value | Range of Factor Values Among Top 50 Parameter Sets | |
|  | | Min | Max |
| **HPV 16** |  |  |  |
| 1-15 | 0.001707 | 1 | 1 |
| 16-27 | 0.002422 | 1 | 1 |
| 28-39 | 0.002577 | 1 | 1 |
| 40-51 | 0.005518 | 1 | 1 |
| 52-60 | 0.014998 | 1 | 1 |
| 61-120 | 0.014998 | 1.09173 | 2.92302 |
| 120+ | 0.014998 | 1.29949 | 4.97673 |
|  |  |  |  |
| **HPV 18** |  |  |  |
| 1-15 | 4.27E-05 | 1 | 1 |
| 16-27 | 0.000189 | 1 | 1 |
| 28-39 | 0.000189 | 1 | 1 |
| 40-51 | 0.007733 | 1 | 1 |
| 52-60 | 0.007733 | 1 | 1 |
| 61-120 | 0.007733 | 1.09173 | 2.92302 |
| 120+ | 0.007733 | 1.29949 | 4.97673 |
|  |  |  |  |
| **HPV 31** |  |  |  |
| 1-15 | 2.62E-04 | 1 | 1 |
| 16-27 | 0.00278007 | 1 | 1 |
| 28-39 | 0.00309101 | 1 | 1 |
| 40-51 | 0.00693095 | 1 | 1 |
| 52-60 | 0.00693095 | 1 | 1 |
| 61-120 | 0.00693095 | 1.09173 | 2.92302 |
| 120+ | 0.00693095 | 1.29949 | 4.97673 |
|  |  |  |  |
| **HPV 33** |  |  |  |
| 1-15 | 7.19E-04 | 1 | 1 |
| 16-27 | 0.00071924 | 1 | 1 |
| 28-39 | 0.00493869 | 1 | 1 |
| 40-51 | 0.00493869 | 1 | 1 |
| 52-60 | 0.00493869 | 1 | 1 |
| 61-120 | 0.00493869 | 1.09173 | 2.92302 |
| 120+ | 0.00493869 | 1.29949 | 4.97673 |
|  |  |  |  |
| **HPV 45** |  |  |  |
| 1-15 | 0.00E+00 | 1 | 1 |
| 16-27 | 0 | 1 | 1 |
| 28-39 | 0.00225755 | 1 | 1 |
| 40-51 | 0.00533318 | 1 | 1 |
| 52-60 | 0.00533318 | 1 | 1 |
| 61-120 | 0.00533318 | 1.09173 | 2.92302 |
| 120+ | 0.00533318 | 1.29949 | 4.97673 |
|  |  |  |  |
| **HPV 52** |  |  |  |
| 1-15 | 8.84E-04 | 1 | 1 |
| 16-27 | 0.00168129 | 1 | 1 |
| 28-39 | 0.00168129 | 1 | 1 |
| 40-51 | 0.00197781 | 1 | 1 |
| 52-60 | 0.00567833 | 1 | 1 |
| 61-120 | 0.00567833 | 1.09173 | 2.92302 |
| 120+ | 0.00567833 | 1.29949 | 4.97673 |
|  |  |  |  |
| **HPV 58** |  |  |  |
| 1-15 | 5.87E-04 | 1 | 1 |
| 16-27 | 0.00246817 | 1 | 1 |
| 28-39 | 0.00246817 | 1 | 1 |
| 40-51 | 0.00461685 | 1 | 1 |
| 52-60 | 0.01024565 | 1 | 1 |
| 61-120 | 0.01024565 | 1.09173 | 2.92302 |
| 120+ | 0.01024565 | 1.29949 | 4.97673 |
|  |  |  |  |
| **High Risk HPV** |  |  |  |
| 1-15 | 1.26E-04 | 1 | 1 |
| 16-27 | 0.00037279 | 1 | 1 |
| 28-39 | 0.0019601 | 1 | 1 |
| 40-51 | 0.0019601 | 1 | 1 |
| 52-60 | 0.0019601 | 1 | 1 |
| 61-120 | 0.0019601 | 1.09173 | 2.92302 |
| 120+ | 0.0019601 | 1.29949 | 4.97673 |
|  |  |  |  |
| **Low Risk HPV** |  |  |  |
| 1-15 | 2.05E-04 | 1 | 1 |
| 16-27 | 0.00029071 | 1 | 1 |
| 28-39 | 0.00030961 | 1 | 1 |
| 40-51 | 0.00066332 | 1 | 1 |
| 52-60 | 0.00066332 | 1 | 1 |
| 61-120 | 0.00066332 | 1.09173 | 2.92302 |
| 120+ | 0.00066332 | 1.29949 | 4.97673 |
|  |  |  | |
| **HPV progression to CIN3** [**^23^**](#_ENREF_23) | | | |
| **HPV Type and Time Since Infection (Months)** | Baseline Value | Range of Factor Values Among Top 50 Parameter Sets | |
|  |  | Min | Max |
| **HPV 16** |  |  |  |
| 1-15 | 3.1611E-05 | 1 | 1 |
| 16-27 | 9.3211E-05 | 1 | 1 |
| 28-39 | 0.00049039 | 1 | 1 |
| 40-51 | 0.00049039 | 1 | 1 |
| 52-60 | 0.00049039 | 1 | 1 |
| 61-120 | 0.00049039 | 1.09173 | 2.92302 |
| 120+ | 0.00049039 | 1.29949 | 4.97673 |
|  |  |  |  |
| **HPV 18** |  |  |  |
| 1-15 | 5.69E-04 | 1 | 1 |
| 16-27 | 0.000808 | 1 | 1 |
| 28-39 | 0.00086 | 1 | 1 |
| 40-51 | 0.001843 | 1 | 1 |
| 52-60 | 0.005024 | 1 | 1 |
| 61-120 | 0.005024 | 1.09173 | 2.92302 |
| 120+ | 0.005024 | 1.29949 | 4.97673 |
|  |  |  |  |
| **HPV 31** |  |  |  |
| 1-15 | 1.07E-05 | 1 | 1 |
| 16-27 | 4.72E-05 | 1 | 1 |
| 28-39 | 4.72E-05 | 1 | 1 |
| 40-51 | 0.001939 | 1 | 1 |
| 52-60 | 0.001939 | 1 | 1 |
| 61-120 | 0.001939 | 1.09173 | 2.92302 |
| 120+ | 0.001939 | 1.29949 | 4.97673 |
|  |  |  |  |
| **HPV 33** |  |  |  |
| 1-15 | 6.55E-05 | 1 | 1 |
| 16-27 | 0.00069574 | 1 | 1 |
| 28-39 | 0.00077365 | 1 | 1 |
| 40-51 | 0.00173726 | 1 | 1 |
| 52-60 | 0.00173726 | 1 | 1 |
| 61-120 | 0.00173726 | 1.09173 | 2.92302 |
| 120+ | 0.00173726 | 1.29949 | 4.97673 |
|  |  |  |  |
| **HPV 45** |  |  |  |
| 1-15 | 1.80E-04 | 1 | 1 |
| 16-27 | 0.00017986 | 1 | 1 |
| 28-39 | 0.00123696 | 1 | 1 |
| 40-51 | 0.00123696 | 1 | 1 |
| 52-60 | 0.00123696 | 1 | 1 |
| 61-120 | 0.00123696 | 1.09173 | 2.92302 |
| 120+ | 0.00123696 | 1.29949 | 4.97673 |
|  |  |  |  |
| **HPV 52** |  |  |  |
| 1-15 | 0.00E+00 | 1 | 1 |
| 16-27 | 0 | 1 | 1 |
| 28-39 | 0.00056487 | 1 | 1 |
| 40-51 | 0.00133597 | 1 | 1 |
| 52-60 | 0.00133597 | 1 | 1 |
| 61-120 | 0.00133597 | 1.09173 | 2.92302 |
| 120+ | 0.00133597 | 1.29949 | 4.97673 |
|  |  |  |  |
| **HPV 58** |  |  |  |
| 1-15 | 2.21E-04 | 1 | 1 |
| 16-27 | 0.00042059 | 1 | 1 |
| 28-39 | 0.00042059 | 1 | 1 |
| 40-51 | 0.00049482 | 1 | 1 |
| 52-60 | 0.00142262 | 1 | 1 |
| 61-120 | 0.00142262 | 1.09173 | 2.92302 |
| 120+ | 0.00142262 | 1.29949 | 4.97673 |
|  |  |  |  |
| **High Risk HPV** |  |  |  |
| 1-15 | 3.16E-05 | 1 | 1 |
| 16-27 | 9.3211E-05 | 1 | 1 |
| 28-39 | 0.00049039 | 1 | 1 |
| 40-51 | 0.00049039 | 1 | 1 |
| 52-60 | 0.00049039 | 1 | 1 |
| 61-120 | 0.00049039 | 1.09173 | 2.92302 |
| 120+ | 0.00049039 | 1.29949 | 4.97673 |
|  |  |  |  |
| **Low Risk HPV** |  |  |  |
| 1-15 | 2.28E-05 | 1 | 1 |
| 16-27 | 3.2305E-05 | 1 | 1 |
| 28-39 | 3.4405E-05 | 1 | 1 |
| 40-51 | 7.3724E-05 | 1 | 1 |
| 52-60 | 7.3724E-05 | 1 | 1 |
| 61-120 | 7.3724E-05 | 1.09173 | 2.92302 |
| 120+ | 7.3724E-05 | 1.29949 | 4.97673 |

| **Progression of CIN2 to Cancer** | | | |
| --- | --- | --- | --- |
| **HPV Type by Duration of Lesion (Years)** | Baseline Value | Range of Factor Values Among Top 50 Parameter Sets | |
|  |  | Min | Max |
| **HPV16** |  |  |  |
| 1-5 | 0.00003294 | 1.00585 | 1.49234 |
| 6-10 | 0.00003564 | 1.00585 | 1.49234 |
| 11-20 | 0.0008568 | 1.00585 | 1.49234 |
| 21-29 | 0.0025056 | 1.00585 | 1.49234 |
| 30-34 | 0.0050112 | 1.00585 | 1.49234 |
| 35-39 | 0.0054288 | 1.00585 | 1.49234 |
| 40-44 | 0.0116928 | 1.00585 | 1.49234 |
| 45-49 | 0.012528 | 1.00585 | 1.49234 |
| 50+ | 0.075 | 1.00585 | 1.49234 |
|  |  |  |  |
| **HPV 18** |  |  |  |
| 1-5 | 0.00003294 | 1.00585 | 1.49234 |
| 6-10 | 3.56E-05 | 1.00585 | 1.49234 |
| 11-20 | 0.0008568 | 1.00585 | 1.49234 |
| 21-29 | 0.0025056 | 1.00585 | 1.49234 |
| 30-34 | 0.0050112 | 1.00585 | 1.49234 |
| 35-39 | 0.0054288 | 1.00585 | 1.49234 |
| 40-44 | 0.0116928 | 1.00585 | 1.49234 |
| 45-49 | 0.012528 | 1.00585 | 1.49234 |
| 50+ | 0.075 | 1.00585 | 1.49234 |
|  |  |  |  |
| **HPV 31** |  |  |  |
| 1-5 | 0.00002196 | 1.00585 | 1.49234 |
| 6-10 | 2.38E-05 | 1.00585 | 1.49234 |
| 11-20 | 0.0005712 | 1.00585 | 1.49234 |
| 21-29 | 0.0016704 | 1.00585 | 1.49234 |
| 30-34 | 0.0033408 | 1.00585 | 1.49234 |
| 35-39 | 0.0036192 | 1.00585 | 1.49234 |
| 40-44 | 0.0077952 | 1.00585 | 1.49234 |
| 45-49 | 0.008352 | 1.00585 | 1.49234 |
| 50+ | 0.05 | 1.00585 | 1.49234 |
|  |  |  |  |
| **HPV 33** |  |  |  |
| 1-5 | 0.00003294 | 1.00585 | 1.49234 |
| 6-10 | 0.0000356 | 1.00585 | 1.49234 |
| 11-20 | 0.0008568 | 1.00585 | 1.49234 |
| 21-29 | 0.0025056 | 1.00585 | 1.49234 |
| 30-34 | 0.0050112 | 1.00585 | 1.49234 |
| 35-39 | 0.0054288 | 1.00585 | 1.49234 |
| 40-44 | 0.0116928 | 1.00585 | 1.49234 |
| 45-49 | 0.012528 | 1.00585 | 1.49234 |
| 50+ | 0.075 | 1.00585 | 1.49234 |
|  |  |  |  |
| **HPV 45** |  |  |  |
| 1-5 | 0.00002196 | 1.00585 | 1.49234 |
| 6-10 | 0.0000238 | 1.00585 | 1.49234 |
| 11-20 | 0.0005712 | 1.00585 | 1.49234 |
| 21-29 | 0.0016704 | 1.00585 | 1.49234 |
| 30-34 | 0.0033408 | 1.00585 | 1.49234 |
| 35-39 | 0.0036192 | 1.00585 | 1.49234 |
| 40-44 | 0.0077952 | 1.00585 | 1.49234 |
| 45-49 | 0.008352 | 1.00585 | 1.49234 |
| 50+ | 0.05 | 1.00585 | 1.49234 |
|  |  |  |  |
| **HPV 52** |  |  |  |
| 1-5 | 0.00002196 | 1.00585 | 1.49234 |
| 6-10 | 0.0000238 | 1.00585 | 1.49234 |
| 11-20 | 0.0005712 | 1.00585 | 1.49234 |
| 21-29 | 0.0016704 | 1.00585 | 1.49234 |
| 30-34 | 0.0033408 | 1.00585 | 1.49234 |
| 35-39 | 0.0036192 | 1.00585 | 1.49234 |
| 40-44 | 0.0077952 | 1.00585 | 1.49234 |
| 45-49 | 0.008352 | 1.00585 | 1.49234 |
| 50+ | 0.05 | 1.00585 | 1.49234 |
|  |  |  |  |
| **HPV 58** |  |  |  |
| 1-5 | 0.00002196 | 1.00585 | 1.49234 |
| 6-10 | 2.38E-05 | 1.00585 | 1.49234 |
| 11-20 | 0.0005712 | 1.00585 | 1.49234 |
| 21-29 | 0.0016704 | 1.00585 | 1.49234 |
| 30-34 | 0.0033408 | 1.00585 | 1.49234 |
| 35-39 | 0.0036192 | 1.00585 | 1.49234 |
| 40-44 | 0.0077952 | 1.00585 | 1.49234 |
| 45-49 | 0.008352 | 1.00585 | 1.49234 |
| 50+ | 0.05 | 1.00585 | 1.49234 |
|  |  |  |  |
| **High Risk HPV** |  |  |  |
| 1-5 | 0.00002196 | 1.00585 | 1.49234 |
| 6-10 | 0.00002376 | 1.00585 | 1.49234 |
| 11-20 | 0.0005712 | 1.00585 | 1.49234 |
| 21-29 | 0.0016704 | 1.00585 | 1.49234 |
| 30-34 | 0.0033408 | 1.00585 | 1.49234 |
| 35-39 | 0.0036192 | 1.00585 | 1.49234 |
| 40-44 | 0.0077952 | 1.00585 | 1.49234 |
| 45-49 | 0.008352 | 1.00585 | 1.49234 |
| 50+ | 0.008352 | 1.00585 | 1.49234 |
|  |  |  |  |
| **Progression of CIN3 to Cancer** | | | |
| **HPV Type by Duration of Lesion (Years)** | Baseline Value | Range of Factor Values Among Top 50 Parameter Sets | |
|  |  | Min | Max |
| **HPV 16** |  |  |  |
| 1-5 | 0.0001647 | 1.01143 | 1.49631 |
| 6-10 | 0.0001782 | 1.01143 | 1.49631 |
| 11-20 | 0.004284 | 1.01143 | 1.49631 |
| 21-29 | 0.012528 | 1.01143 | 1.49631 |
| 30-34 | 0.025056 | 1.01143 | 1.49631 |
| 35-39 | 0.027144 | 1.01143 | 1.49631 |
| 40-44 | 0.058464 | 1.01143 | 1.49631 |
| 45-49 | 0.06264 | 1.01143 | 1.49631 |
| 50+ | 0.075 | 1.01143 | 1.49631 |
|  |  |  |  |
| **HPV 18** |  |  |  |
| 1-5 | 0.0001647 | 1.01143 | 1.49631 |
| 6-10 | 0.0001782 | 1.01143 | 1.49631 |
| 11-20 | 0.004284 | 1.01143 | 1.49631 |
| 21-29 | 0.012528 | 1.01143 | 1.49631 |
| 30-34 | 0.025056 | 1.01143 | 1.49631 |
| 35-39 | 0.027144 | 1.01143 | 1.49631 |
| 40-44 | 0.058464 | 1.01143 | 1.49631 |
| 45-49 | 0.06264 | 1.01143 | 1.49631 |
| 50+ | 0.075 | 1.01143 | 1.49631 |
|  |  |  |  |
| **HPV 31** |  |  |  |
| 1-5 | 0.0001098 | 1.01143 | 1.49631 |
| 6-10 | 0.0001188 | 1.01143 | 1.49631 |
| 11-20 | 0.002856 | 1.01143 | 1.49631 |
| 21-29 | 0.008352 | 1.01143 | 1.49631 |
| 30-34 | 0.016704 | 1.01143 | 1.49631 |
| 35-39 | 0.018096 | 1.01143 | 1.49631 |
| 40-44 | 0.038976 | 1.01143 | 1.49631 |
| 45-49 | 0.04176 | 1.01143 | 1.49631 |
| 50+ | 0.05 | 1.01143 | 1.49631 |
|  |  |  |  |
| **HPV 33** |  |  |  |
| 1-5 | 0.0001647 | 1.01143 | 1.49631 |
| 6-10 | 0.0001782 | 1.01143 | 1.49631 |
| 11-20 | 0.004284 | 1.01143 | 1.49631 |
| 21-29 | 0.012528 | 1.01143 | 1.49631 |
| 30-34 | 0.025056 | 1.01143 | 1.49631 |
| 35-39 | 0.027144 | 1.01143 | 1.49631 |
| 40-44 | 0.058464 | 1.01143 | 1.49631 |
| 45-49 | 0.06264 | 1.01143 | 1.49631 |
| 50+ | 0.075 | 1.01143 | 1.49631 |
|  |  |  |  |
| **HPV 45** |  |  |  |
| 1-5 | 0.0001098 | 1.01143 | 1.49631 |
| 6-10 | 0.0001188 | 1.01143 | 1.49631 |
| 11-20 | 0.002856 | 1.01143 | 1.49631 |
| 21-29 | 0.008352 | 1.01143 | 1.49631 |
| 30-34 | 0.016704 | 1.01143 | 1.49631 |
| 35-39 | 0.018096 | 1.01143 | 1.49631 |
| 40-44 | 0.038976 | 1.01143 | 1.49631 |
| 45-49 | 0.04176 | 1.01143 | 1.49631 |
| 50+ | 0.05 | 1.01143 | 1.49631 |
|  |  |  |  |
| **HPV 52** |  |  |  |
| 1-5 | 0.0001098 | 1.01143 | 1.49631 |
| 6-10 | 0.0001188 | 1.01143 | 1.49631 |
| 11-20 | 0.002856 | 1.01143 | 1.49631 |
| 21-29 | 0.008352 | 1.01143 | 1.49631 |
| 30-34 | 0.016704 | 1.01143 | 1.49631 |
| 35-39 | 0.018096 | 1.01143 | 1.49631 |
| 40-44 | 0.038976 | 1.01143 | 1.49631 |
| 45-49 | 0.04176 | 1.01143 | 1.49631 |
| 50+ | 0.05 | 1.01143 | 1.49631 |
|  |  |  |  |
| **HPV 58** |  |  |  |
| 1-5 | 0.0001098 | 1.01143 | 1.49631 |
| 6-10 | 0.0001188 | 1.01143 | 1.49631 |
| 11-20 | 0.002856 | 1.01143 | 1.49631 |
| 21-29 | 0.008352 | 1.01143 | 1.49631 |
| 30-34 | 0.016704 | 1.01143 | 1.49631 |
| 35-39 | 0.018096 | 1.01143 | 1.49631 |
| 40-44 | 0.038976 | 1.01143 | 1.49631 |
| 45-49 | 0.04176 | 1.01143 | 1.49631 |
| 50+ | 0.05 | 1.01143 | 1.49631 |
|  |  |  |  |
| **High Risk HPV** |  |  |  |
| 1-5 | 0.0001098 | 1.01143 | 1.49631 |
| 6-10 | 0.0001188 | 1.01143 | 1.49631 |
| 11-20 | 0.002856 | 1.01143 | 1.49631 |
| 21-29 | 0.008352 | 1.01143 | 1.49631 |
| 30-34 | 0.016704 | 1.01143 | 1.49631 |
| 35-39 | 0.018096 | 1.01143 | 1.49631 |
| 40-44 | 0.038976 | 1.01143 | 1.49631 |
| 45-49 | 0.04176 | 1.01143 | 1.49631 |
| 50+ | 0.04176 | 1.01143 | 1.49631 |

| **Progression of invasive cancer stages** [**^5^**](#_ENREF_5)**^,^**[**^6^**](#_ENREF_6) | | | | | |
| --- | --- | --- | --- | --- | --- |
|  | | Baseline Value | | | |
| Local to regional | | 0.02 | | | |
| Regional to distant | | 0.025 | | | |
|  | |  | | | |
| **Probability of symptom detection** [**^5^**](#_ENREF_5)**^,^**[**^6^**](#_ENREF_6) | |  | | | |
|  | | Baseline Value | | | |
| Local | | 0.0039 | | | |
| Regional | | 0.1333 | | | |
| Distant | | 0.1746 | | | |
|  | |  | | | |
| **Invasive cancer mortality, undetected cancer** [**^24^**](#_ENREF_24)**^,^**[**^25^**](#_ENREF_25) | |  | | | |
| **Stage and Duration, years** | | Baseline Value | | | |
| Local | | 0.008683 | | | |
| Regional | | 0.020897 | | | |
| Distant | | 0.072469 | | | |
|  | |  | | | |
| **Invasive cancer mortality, detected cancer** [**^6^**](#_ENREF_6)**^,^**[**^24^**](#_ENREF_24) | |  | | | |
| **Stage and Duration, years** | | Baseline Value | | | |
| **Local** | |  | | | |
| 1 | | 0.009276 | | | |
| 2 | | 0.011942 | | | |
| 3-10 | | 0.004832 | | | |
| 10+ | | 0 | | | |
|  | |  | | | |
| **Regional** | |  | | | |
| 1 | | 0.020449 | | | |
| 2 | | 0.026715 | | | |
| 3-10 | | 0.015525 | | | |
| 10+ | | 0 | | | |
|  | |  | | | |
| **Distant** | |  | | | |
| 1 | | 0.064418 | | | |
| 2 | | 0.08563 | | | |
| 3-10 | | 0.06736 | | | |
| 10+ | | 0 | | | |
|  | |  | | | |
| **HPV clearance** [**^3^**](#_ENREF_3)**^,^**[**^16-19^**](#_ENREF_16)**^,^**[**^22^**](#_ENREF_22)**^,^**[**^23^**](#_ENREF_23)**^,^**[**^26-28^**](#_ENREF_26) | | | | |  |
| **HPV Type by Time Since Infection (Months)** | Baseline Value | | Range of Factor Values Among Top 50 Parameter Sets | |  |
|  |  | | Min | Max |  |
| **HPV 16** |  | |  |  |  |
| 1-15 | 0.041886 | | 0.526718 | 0.862163 |  |
| 16-27 | 0.040754 | | 0.526718 | 0.862163 |  |
| 28-39 | 0.033905 | | 0.526718 | 0.862163 |  |
| 40-63 | 0.031888 | | 0.526718 | 0.862163 |  |
| 64+ | 0.019846 | | 0.526718 | 0.862163 |  |
|  |  | |  |  |  |
| **HPV 18** |  | |  |  |  |
| 1-15 | 0.073342 | | 0.526718 | 0.862163 |  |
| 16-27 | 0.063235 | | 0.526718 | 0.862163 |  |
| 28-39 | 0.053605 | | 0.526718 | 0.862163 |  |
| 40-63 | 0.020616 | | 0.526718 | 0.862163 |  |
| 64+ | 0.020616 | | 0.526718 | 0.862163 |  |
|  |  | |  |  |  |
| **HPV 31** |  | |  |  |  |
| 1-15 | 0.063447 | | 0.526718 | 0.862163 |  |
| 16-27 | 0.033826 | | 0.526718 | 0.862163 |  |
| 28-39 | 0.033826 | | 0.526718 | 0.862163 |  |
| 40-63 | 0.033826 | | 0.526718 | 0.862163 |  |
| 64+ | 0.033826 | | 0.526718 | 0.862163 |  |
|  |  | |  |  |  |
| **HPV 33** |  | |  |  |  |
| 1-15 | 0.083452 | | 0.526718 | 0.862163 |  |
| 16-27 | 0.044955 | | 0.526718 | 0.862163 |  |
| 28-39 | 0.036156 | | 0.526718 | 0.862163 |  |
| 40-63 | 0.036156 | | 0.526718 | 0.862163 |  |
| 64+ | 0.036156 | | 0.526718 | 0.862163 |  |
|  |  | |  |  |  |
| **HPV 45** |  | |  |  |  |
| 1-15 | 0.078517 | | 0.526718 | 0.862163 |  |
| 16-27 | 0.042579 | | 0.526718 | 0.862163 |  |
| 28-39 | 0.041675 | | 0.526718 | 0.862163 |  |
| 40-49 | 0.030133 | | 0.526718 | 0.862163 |  |
| 50+ | 0.01507 | | 0.526718 | 0.862163 |  |
|  |  | |  |  |  |
| **HPV 52** |  | |  |  |  |
| 1-15 | 0.062999 | | 0.526718 | 0.862163 |  |
| 16-27 | 0.044401 | | 0.526718 | 0.862163 |  |
| 28-39 | 0.044401 | | 0.526718 | 0.862163 |  |
| 40-51 | 0.039325 | | 0.526718 | 0.862163 |  |
| 52+ | 0.039325 | | 0.526718 | 0.862163 |  |
|  |  | |  |  |  |
| **HPV 58** |  | |  |  |  |
| 1-15 | 0.065572 | | 0.526718 | 0.862163 |  |
| 16-27 | 0.05443 | | 0.526718 | 0.862163 |  |
| 28-39 | 0.053968 | | 0.526718 | 0.862163 |  |
| 40-51 | 0.033319 | | 0.526718 | 0.862163 |  |
| 52+ | 0.01666 | | 0.526718 | 0.862163 |  |
|  |  | |  |  |  |
| **High Risk HPV** |  | |  |  |  |
| 1-15 | 0.080766 | | 0.526718 | 0.862163 |  |
| 16-27 | 0.066633 | | 0.526718 | 0.862163 |  |
| 28-39 | 0.053972 | | 0.526718 | 0.862163 |  |
| 40-63 | 0.049229 | | 0.526718 | 0.862163 |  |
| 64+ | 0.005094 | | 0.526718 | 0.862163 |  |
|  |  | |  |  |  |
| **Low Risk HPV** |  | |  |  |  |
| 1-15 | 0.051888 | | 0.526718 | 0.862163 |  |
| 16-27 | 0.050005 | | 0.526718 | 0.862163 |  |
| 28-39 | 0.034649 | | 0.526718 | 0.862163 |  |
| 40-51 | 0.034649 | | 0.526718 | 0.862163 |  |
| 52-63 | 0.028608 | | 0.526718 | 0.862163 |  |
| 64+ | 0.041886 | | 0.526718 | 0.862163 |  |

| **Regression of CIN2 to Healthy** | | | | | |
| --- | --- | --- | --- | --- | --- |
| **HPV Type by Duration of Lesion(Years)** | Baseline Value | | | Range of Factor Values Among Top 50 Parameter Sets | |
|  |  | | | Min | Max |
| **HPV 16** |  | | |  |  |
| 1-5 | 0.045 | | | 0.502265 | 3.94165 |
| 6-10 | 0.036 | | | 0.502265 | 3.94165 |
| 11-20 | 0.027 | | | 0.502265 | 3.94165 |
| 21-29 | 0.0018 | | | 0.502265 | 3.94165 |
| 30-39 | 0.0009 | | | 0.502265 | 3.94165 |
| 40+ | 0.00045 | | | 0.502265 | 3.94165 |
|  |  | |  |  |  |
| **All Other HPV Types** |  | |  |  |  |
| 1-5 | 0.05 | | | 0.707622 | 3.99684 |
| 6-10 | 0.04 | | | 0.707622 | 3.99684 |
| 11-20 | 0.03 | | | 0.707622 | 3.99684 |
| 21-29 | 0.002 | | | 0.707622 | 3.99684 |
| 30-39 | 0.001 | | | 0.707622 | 3.99684 |
| 40+ | 0.0005 | | | 0.707622 | 3.99684 |
|  | | | | | |
| **Regression of CIN3 to Healthy** | | | | | |
| **HPV Type by Duration of Lesion (Years)** | Baseline Value | | | Range of Factor Values Among Top 50 Parameter Sets | |
|  |  | | | Min | Max |
| **HPV 16** |  | |  |  |  |
| 1-5 | 0.0225 | | | 0.505983 | 3.96022 |
| 6-10 | 0.018 | | | 0.505983 | 3.96022 |
| 11-20 | 0.0135 | | | 0.505983 | 3.96022 |
| 21-29 | 0.0009 | | | 0.505983 | 3.96022 |
| 30-39 | 0.00045 | | | 0.505983 | 3.96022 |
| 40+ | 0.000225 | | | 0.505983 | 3.96022 |
|  |  | |  |  |  |
| **All Other HPV Types** | |  |  |  |  |
| 1-5 | 0.025 | | | 0.627965 | 3.96406 |
| 6-10 | 0.02 | | | 0.627965 | 3.96406 |
| 11-20 | 0.015 | | | 0.627965 | 3.96406 |
| 21-29 | 0.001 | | | 0.627965 | 3.96406 |
| 30-39 | 0.0005 | | | 0.627965 | 3.96406 |
| 40+ | 0.00025 | | | 0.627965 | 3.96406 |

^a^ A range (minimum, maximum) of values is presented for the baseline transition probability between Healthy and HPV infected because transitions vary between single age years; the range represents the minimum and maximum value within an age group.

**Supplementary Table 2. HIV natural history parameters and assumptions.**

| Age at HIV infection | 20 years^[29](#_ENREF_29)^ |
| --- | --- |
| CD4+ count following HIV infection | 600 cells/ul^[30](#_ENREF_30)^ |
| Decline in CD4+ count per month in absence of ART, by CD4+ level | CD4+>500 cells/ul: 5.9  CD4+ 351-500 cells/ul: 3.8  CD4+ 201-350 cells/ul: 2.6 ^[30](#_ENREF_30),[31](#_ENREF_31)^ |
| Monthly probability of HIV death in absence of ART, by CD4+ level | >350 cells/ul: 0.008  201-350 cells/ul: 0.011 |
| CD4+ count at HIV presentation/ART initiation | 350 cells/ul (age 25 years)^[32](#_ENREF_32)^ |
| Relative survival model for excess mortality due to HIV for women receiving ART, based on initiating ART at age 25 years with a CD4+ count >200 cells/ul; survival varies by years since ART initiation | ^[33](#_ENREF_33)^ |

**Supplementary Table 3. Costs of cancer treatment, US$ 2017.**^[34-39](#_ENREF_34)^

| **Cancer stage and procedures** | **Proportion of women in given stage receiving procedure** | **Direct Medical Cost** | **Cost of women’s time, transportation, and follow-up visits** |
| --- | --- | --- | --- |
| *Staging* |  |  | *28.62* |
| Vaginal and rectal exam | 100% | 18.88 |  |
| Abdominal ultrasound | 100% | 45.71 |  |
| HIV rapid screen test | 40% | 1.27 |  |
| Chest X-ray | 100% | 45.71 |  |
| Full blood count | 100% | 4.26 |  |
| Urea and electrolytes | 100% | 14.82 |  |
| Liver function test | 100% | 18.19 |  |
| Cystoscopy | 100% | 86.12 |  |
| *Stage 1a1* |  |  | *106.97* |
| Hysterectomy - simple | 100% | 363.63 |  |
| *Stage 1a2* |  |  | *148.16* |
| Hysterectomy - radical | 100% | 1,454.45 |  |
| *Stage 1b1* |  |  | *148.16* |
| Hysterectomy - radical | 100% | 1,454.45 |  |
| *Stage 1b2* |  |  | *529.81* |
| CT planning | 100% | 1,643.77 |  |
| Radiotherapy | 100% | 4,567.22 |  |
| Chemotherapy 1 | 100% | 1,442.16 |  |
| Brachytherapy | 100% | 930.73 |  |
| *Stage 2a* |  |  | *338.98* |
| Hysterectomy - radical | 50% | 727.23 |  |
| CT planning | 50% | 821.88 |  |
| Radiotherapy | 50% | 2,283.61 |  |
| Chemotherapy 1 | 50% | 721.08 |  |
| Brachytherapy | 50% | 465.36 |  |
| *Stage 2b* |  |  | *529.81* |
| CT planning | 100% | 1,643.77 |  |
| Radiotherapy | 100% | 4,567.22 |  |
| Chemotherapy 1 | 100% | 1,442.16 |  |
| Brachytherapy | 100% | 930.73 |  |
| *Stage 3a* |  |  | *529.81* |
| CT planning | 100% | 1,643.77 |  |
| Radiotherapy | 100% | 4,567.22 |  |
| Chemotherapy 1 | 100% | 1,442.16 |  |
| Brachytherapy | 100% | 930.73 |  |
| *Stage 3b* |  |  | *529.81* |
| CT planning | 100% | 1,643.77 |  |
| Radiotherapy | 100% | 4,567.22 |  |
| Chemotherapy 1 | 100% | 1,442.16 |  |
| Brachytherapy | 100% | 930.73 |  |
| *Stage 4a* |  |  | *529.81* |
| CT planning | 100% | 1,643.77 |  |
| Radiotherapy | 100% | 4,567.22 |  |
| Chemotherapy 1 | 100% | 1,442.16 |  |
| Brachytherapy | 100% | 930.73 |  |
| *Stage 4b* |  |  | *505.68* |
| CT planning | 100% | 1,643.77 |  |
| Radiotherapy | 100% | 4,567.22 |  |
| Chemotherapy 2 | 100% | 1,882.26 |  |
| Brachytherapy | 50% | 465.36 |  |

**Supplementary Figure 1. Model fit to epidemiologic data on the burden of HPV among HIV-infected women: age-specific prevalence of oncogenic HPV.** Model output from the 50 top-fitting input parameter sets following likelihood-based scoring is displayed by gray circles, while the 95% confidence intervals from empirical data are represented by the black lines for age-specific prevalence of oncogenic HPV in HIV-infected women.[^40^](#_ENREF_40)

**
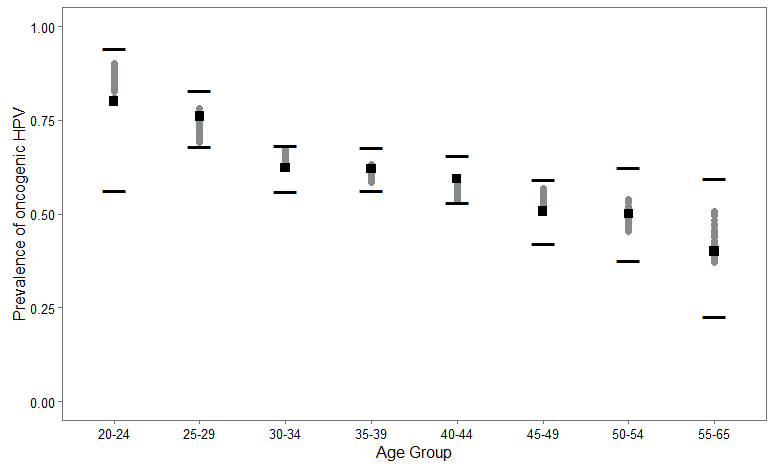
**

**Supplementary Figure 2. Model fit to epidemiologic data on the burden of HPV among HIV-infected women: HPV genotype distribution in cervical intraepithelial neoplasia grade 3 (CIN3) and cervical cancer.** Model output from the 50 top-fitting input parameter sets following likelihood-based scoring is displayed by gray circles, while the 95% confidence intervals from empirical data are represented by the black lines for HPV genotype distribution in cervical intraepithelial neoplasia grade 3 (CIN3)[^41^](#_ENREF_41) and cervical cancer. [^42^](#_ENREF_42)

**
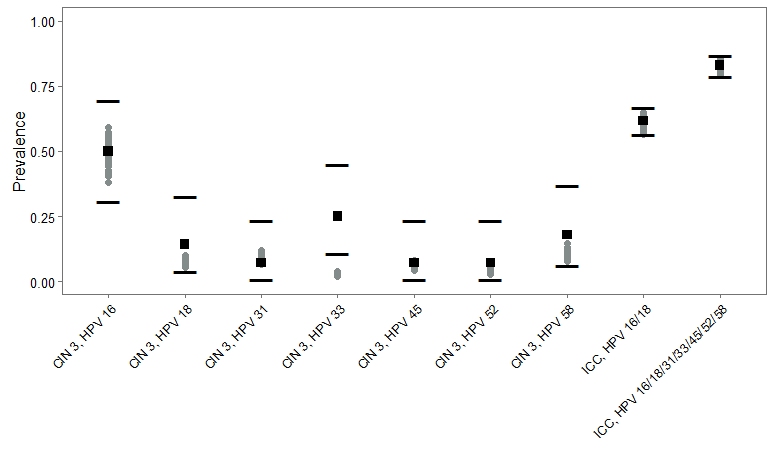
**

**Supplementary Figure 3. Model validation: prevalence of CIN2/3 in HIV-infected women in South Africa.** Black squares represent empirical data from a study of HIV-infected women in Cape Town, South Africa ^[43](#_ENREF_43)^; gray circles represent model output from the 50 top-fitting input parameter sets.

**
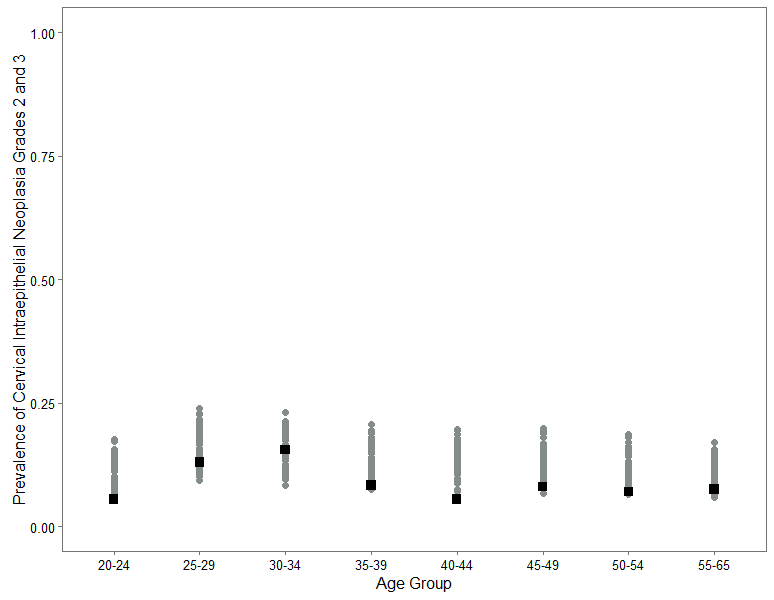
**

**Supplementary Figure 4. Model validation: age-specific cervical cancer incidence in South Africa.** Black lines represent empirical data from the National Cancer Registry in South Africa (general population), 2011 [^44^](#_ENREF_44). Gray dots represent model outputted cervical cancer incidence from the 50 top-fitting parameter sets for women infected with HIV at age 20 years. Data on cervical cancer incidence in HIV-infected women are limited, but hazard ratios for HIV-infected versus HIV-uninfected women range from 5.8 (2.3-14.6) in women with a CD4 cell count between 200 and 349 cells/µl and 1.7 (0.9-3.2) for women with a CD4 cell count greater than 350 cells/µl in the North American AIDS Cohort Collaboration on Research and Design Cohort (NA-ACCORD) ^[45](#_ENREF_45)^. A study of South African cohorts with HIV found that the incidence of cervical cancer in HIV-infected women was nearly 500 per 100,000 person-years [^46^](#_ENREF_46). Model outputs for cancer incidence appear to be consistent with this wide range of cancer incidence suggested by the literature.

**
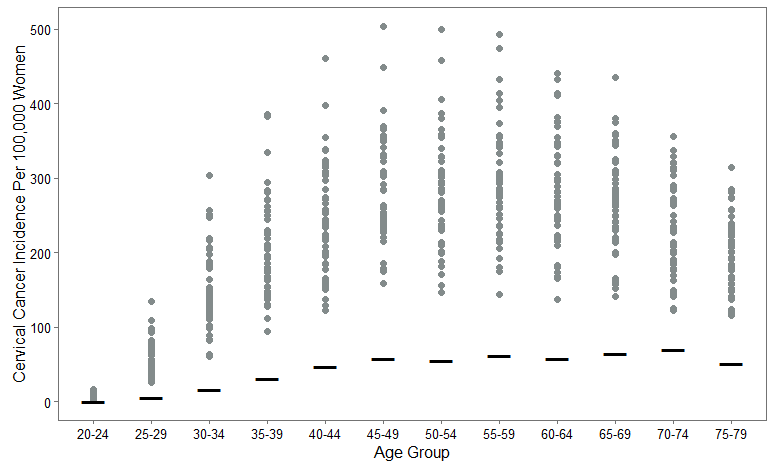
**

**Supplementary Figure 5. Screening and management algorithm: Pap with ASCUS+ referral threshold (Pap ASCUS+).**

**
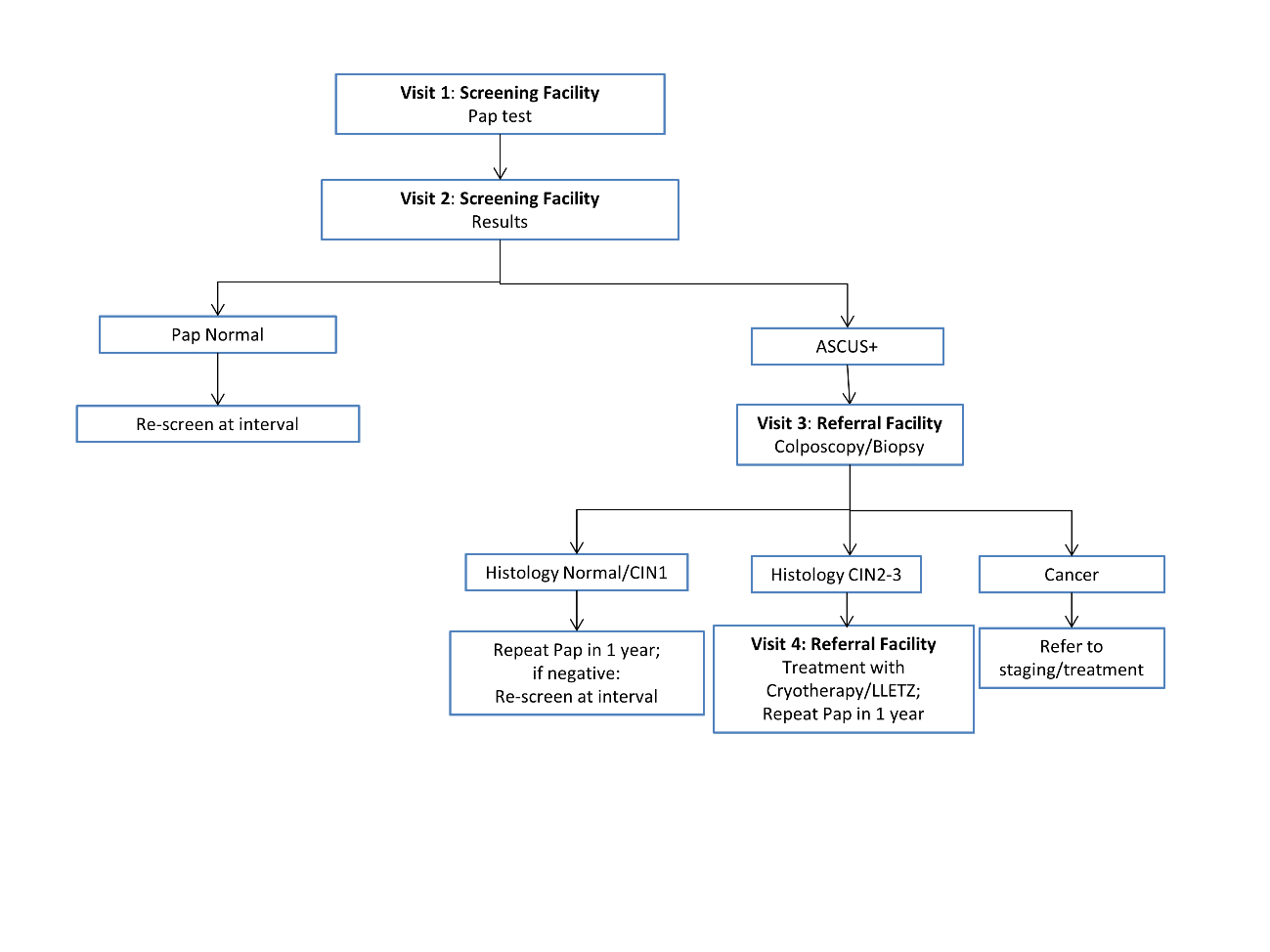
**

**Supplementary Figure 6. Screening and management algorithm: Pap with ASC-H/HSIL+ referral threshold (Pap HSIL+).**

**
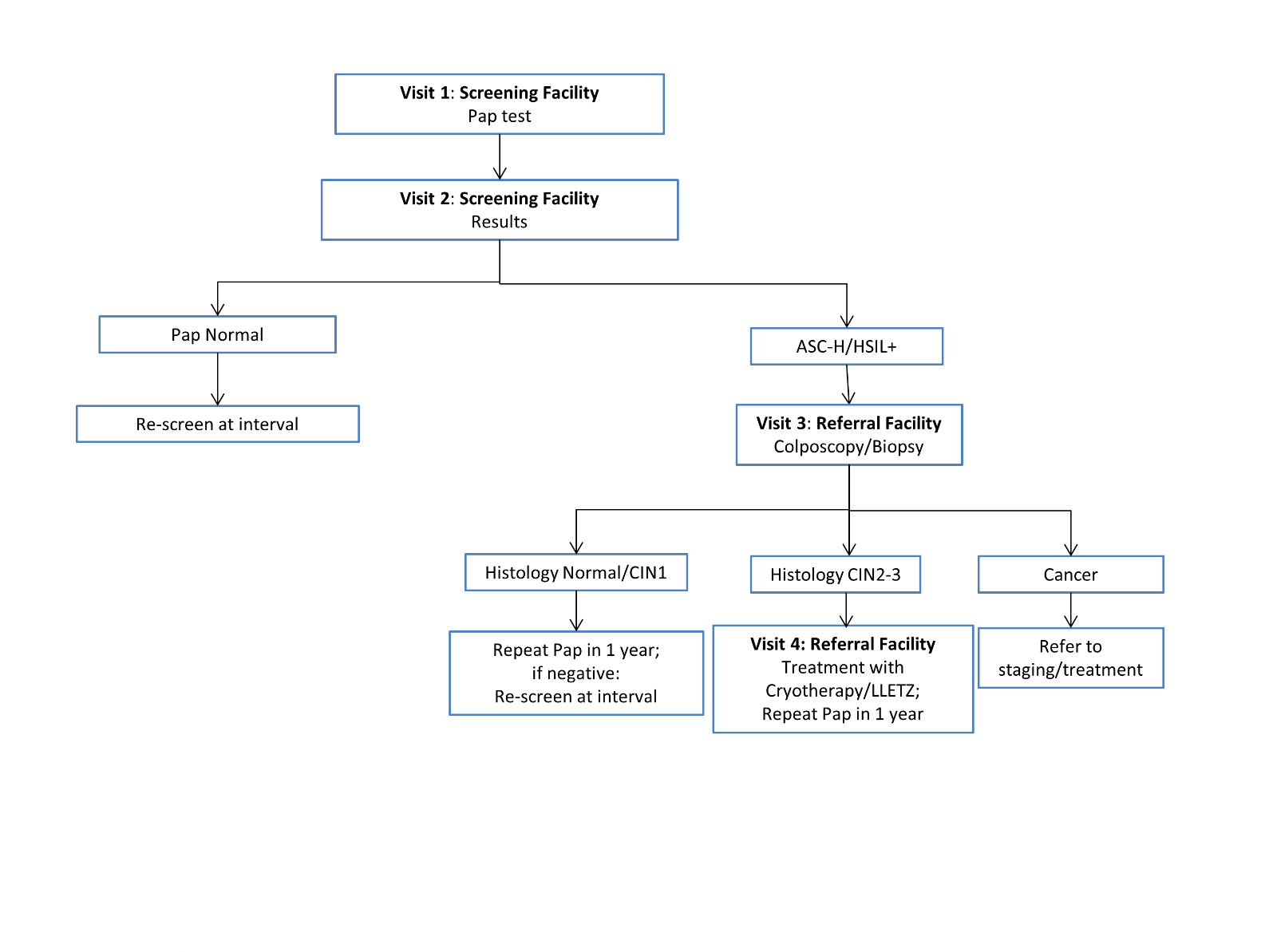
**

**Supplementary Figure 7. Screening and management algorithm: HPV test-and-treat.**

**
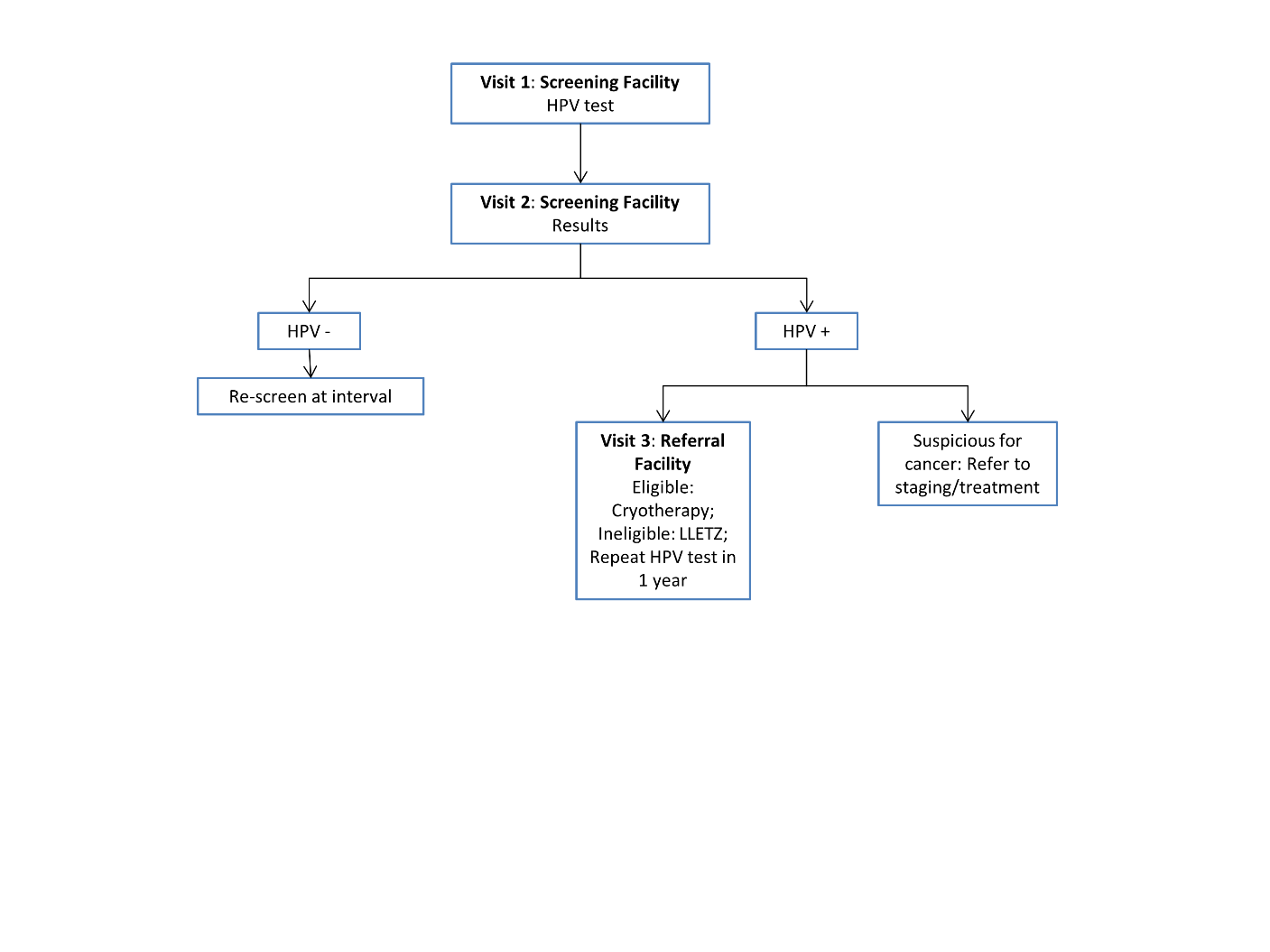
**

**Supplementary Figure 8. Screening and management algorithm: HPV with visual inspection with acetic acid (VIA) triage of HPV-positive women (HPV-VIA).**

**
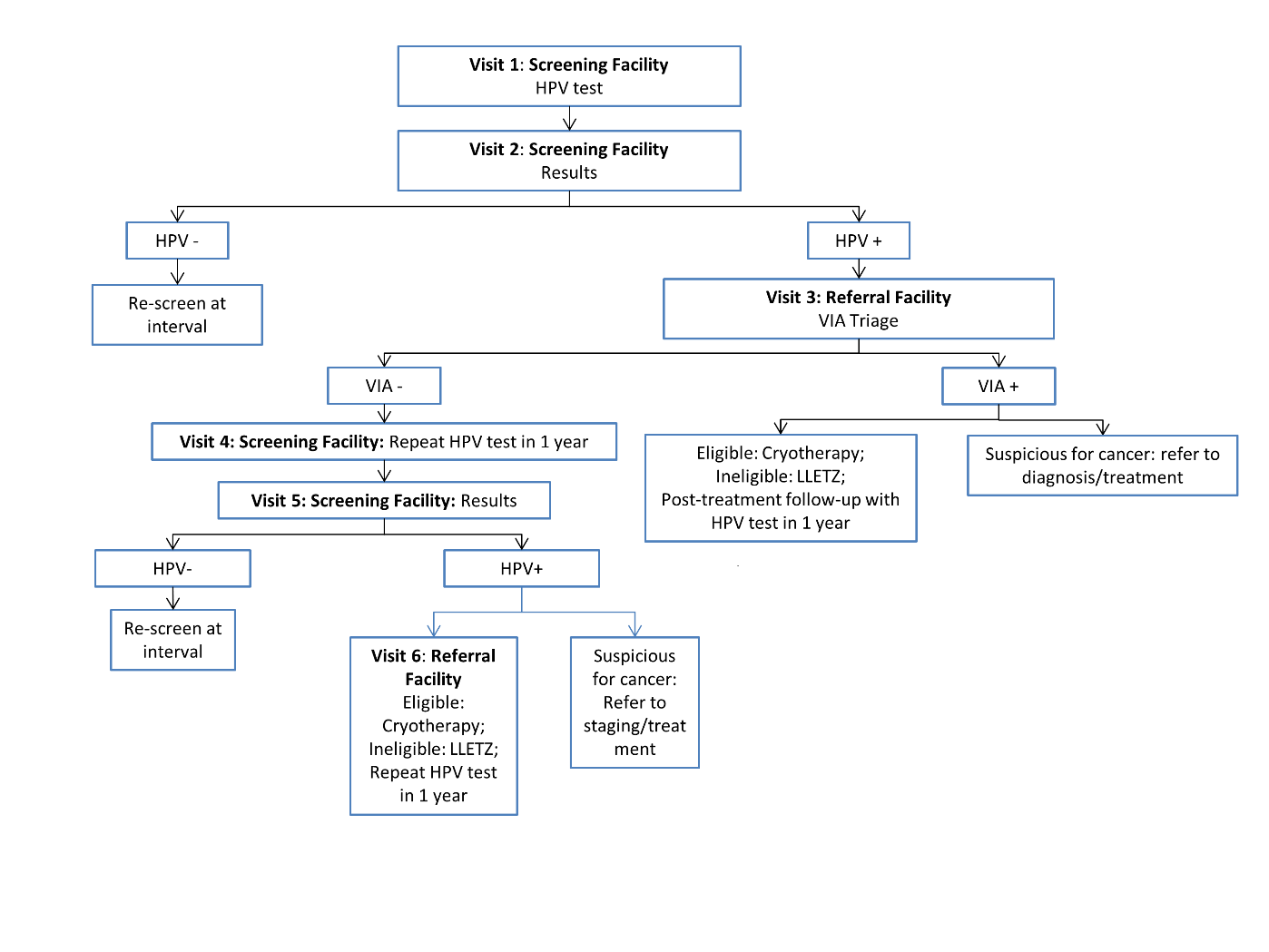
**

**Supplementary Figure 9. Screening and management algorithm: HPV with Pap triage of HPV-positive women (HPV-Pap).**

**
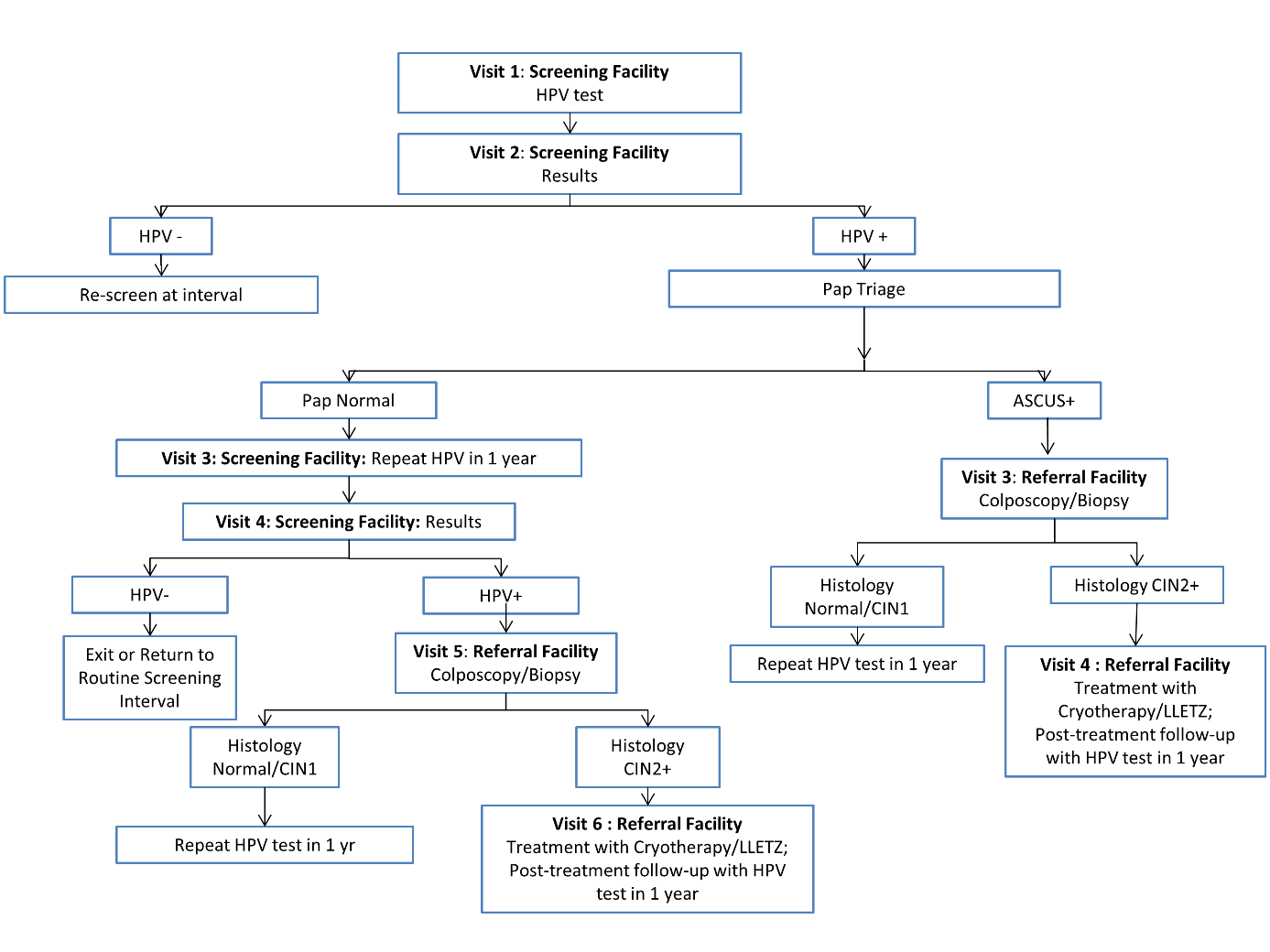
**

**Supplementary Figure 10. Screening and management algorithm: HPV testing with HPV16/18 genotyping (HPV 16/18 genotyping).**

**
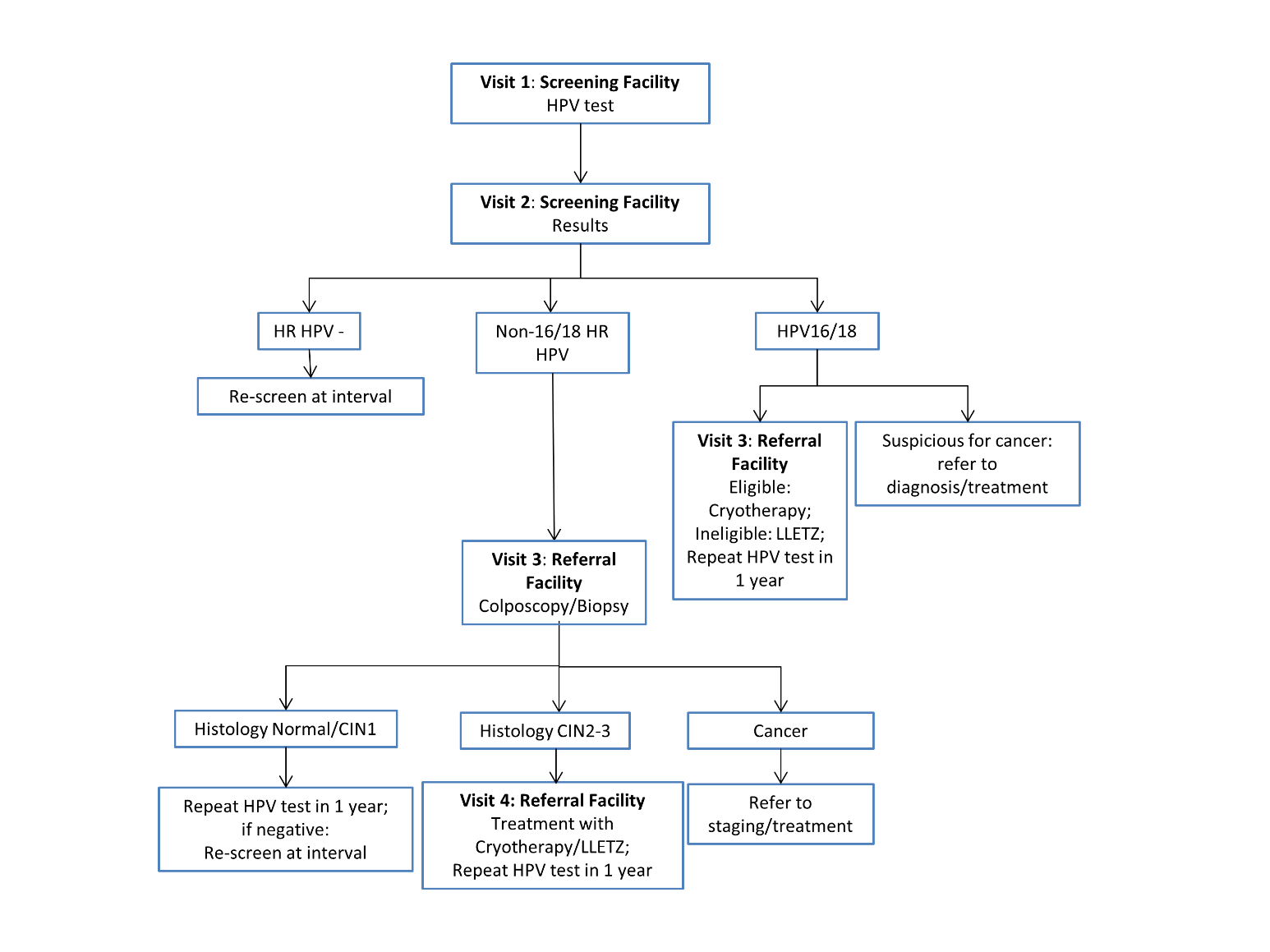
**

**Supplementary Figure 11. Screening and management algorithm: Visual inspection with acetic acid (VIA).** VIA was assumed to occur for 15% of the population under all other screening strategies. Costs and health outcomes were weighted accordingly.


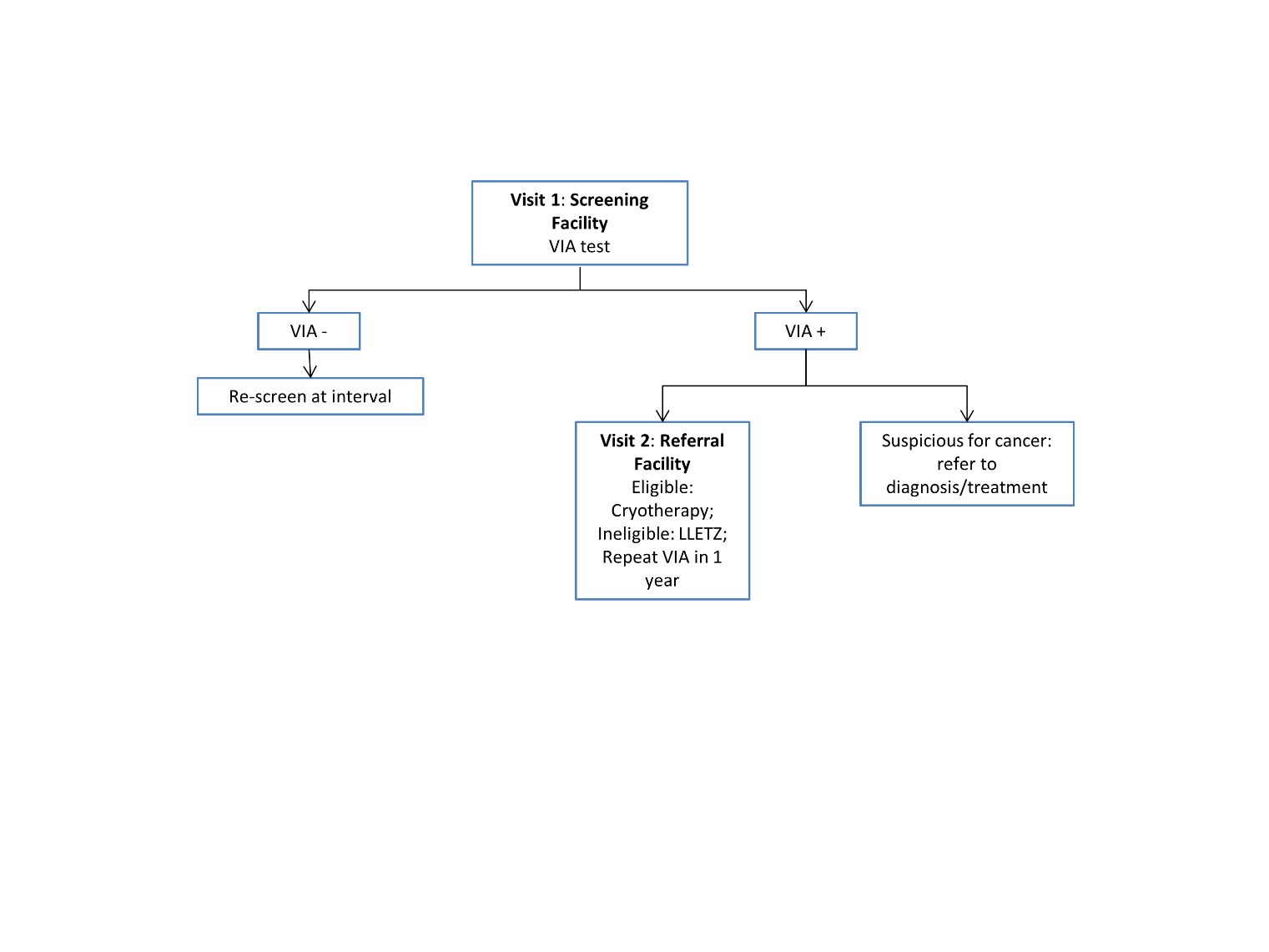


**References**

**1.** Campos NG, Burger EA, Sy S, et al. An updated natural history model of cervical cancer: derivation of model parameters. *Am J Epidemiol.* 2014;180:545-555.

**2.** Campos NG, Tsu, V., Jeronimo, J., Mvundura, M., Lee, K., Kim, J.J. When and how often to screen for cervical cancer in three low- and middle-income countries: A cost-effectiveness analysis. *Papillomavirus Research.* 2015;1:38-58.

**3.** Adler D, Wallace M, Bennie T, et al. High risk human papillomavirus persistence among HIV-infected young women in South Africa. *Int J Infect Dis.* 2015;33:219-221.

**4.** Ahdieh L, Munoz A, Vlahov D, et al. Cervical neoplasia and repeated positivity of human papillomavirus infection in human immunodeficiency virus-seropositive and -seronegative women. *Am J Epidemiol.* 2000;151:1148-1157.

**5.** Denny L, Adewole I, Anorlu R, et al. Human papillomavirus prevalence and type distribution in invasive cervical cancer in sub-Saharan Africa. *Int J Cancer.* 2014;134:1389-1398.

**6.** Dryden-Peterson S, Bvochora-Nsingo M, Suneja G, et al. HIV Infection and Survival Among Women With Cervical Cancer. *J Clin Oncol.* 2016.

**7.** International Monetary Fund. World Economic Outlook Database. 2014.

**8.** Lince-Deroche N, Phiri J, Michelow P, et al. Costs and Cost Effectiveness of Three Approaches for Cervical Cancer Screening among HIV-Positive Women in Johannesburg, South Africa. *PLoS One.* 2015;10:e0141969.

**9.** Lince-Deroche N, Van Rensberg C, Phiri J, et al. Cost-effectiveness of 2 methods to treat cervical dysplasia in HIV-positive women. Paper presented at: Conference on Retroviruses and Opportunistic Infections; February 13-16, 2017, 2017; Seattle, Washington.

**10.** Lince-Deroche N. Personal communication.

**11.** South African National Department of Labor. Domestic worker minimum wage increases from 1 December 2016. <http://www.labour.gov.za/DOL/downloads/legislation/sectoral-determinations/basic-conditions-of-employment/domesticwages2016_2017.pdf> Accessed April 28, 2017.

**12.** ILOSTAT. <http://www.ilo.org/ilostat>. Accessed March 15, 2017.

**13.** Meyer-Rath G. National ART Cost Model, South Africa. Health Economics and Epidemiology Research Office, Boston University/University of the Witwatersrand, Johannesburg.2017.

**14.** Meyer-Rath G, Brennan AT, Fox MP, et al. Rates and cost of hospitalization before and after initiation of antiretroviral therapy in urban and rural settings in South Africa. *J Acquir Immune Defic Syndr.* 2013;62:322-328.

**15.** Munoz N, Mendez F, Posso H, et al. Incidence, duration, and determinants of cervical human papillomavirus infection in a cohort of Colombian women with normal cytological results. *J Infect Dis.* 2004;190:2077-2087.

**16.** Banura C, Sandin S, van Doorn LJ, et al. Type-specific incidence, clearance and predictors of cervical human papillomavirus infections (HPV) among young women: a prospective study in Uganda. *Infect Agent Cancer.* 2010;5:7.

**17.** Blitz S, Baxter J, Raboud J, et al. Evaluation of HIV and highly active antiretroviral therapy on the natural history of human papillomavirus infection and cervical cytopathologic findings in HIV-positive and high-risk HIV-negative women. *J Infect Dis.* 2013;208:454-462.

**18.** Ahdieh L, Klein RS, Burk R, et al. Prevalence, incidence, and type-specific persistence of human papillomavirus in human immunodeficiency virus (HIV)-positive and HIV-negative women. *J Infect Dis.* 2001;184:682-690.

**19.** Mbulawa ZZ, Marais DJ, Johnson LF, et al. Impact of human immunodeficiency virus on the natural history of human papillomavirus genital infection in South African men and women. *J Infect Dis.* 2012;206:15-27.

**20.** Minkoff H, Feldman J, DeHovitz J, et al. A longitudinal study of human papillomavirus carriage in human immunodeficiency virus-infected and human immunodeficiency virus-uninfected women. *Am J Obstet Gynecol.* 1998;178:982-986.

**21.** Safaeian M, Kiddugavu M, Gravitt PE, et al. Determinants of incidence and clearance of high-risk human papillomavirus infections in rural Rakai, Uganda. *Cancer Epidemiol Biomarkers Prev.* 2008;17:1300-1307.

**22.** Strickler HD, Burk RD, Fazzari M, et al. Natural history and possible reactivation of human papillomavirus in human immunodeficiency virus-positive women. *J Natl Cancer Inst.* 2005;97:577-586.

**23.** Herrero R, Wacholder S, Rodriguez AC, et al. Prevention of persistent human papillomavirus infection by an HPV16/18 vaccine: a community-based randomized clinical trial in Guanacaste, Costa Rica. *Cancer Discov.* 2011;1:408-419.

**24.** Coghill AE, Shiels MS, Suneja G, et al. Elevated Cancer-Specific Mortality Among HIV-Infected Patients in the United States. *J Clin Oncol.* 2015;33:2376-2383.

**25.** Sankaranarayanan R, Swaminathan R, Brenner H, et al. Cancer survival in Africa, Asia, and Central America: a population-based study. *Lancet Oncol.* 2010;11:165-173.

**26.** Moscicki AB, Ellenberg JH, Farhat S, et al. Persistence of human papillomavirus infection in HIV-infected and -uninfected adolescent girls: risk factors and differences, by phylogenetic type. *J Infect Dis.* 2004;190:37-45.

**27.** Koshiol J, Schroeder J, Jamieson DJ, et al. Smoking and time to clearance of human papillomavirus infection in HIV-seropositive and HIV-seronegative women. *Am J Epidemiol.* 2006;164:176-183.

**28.** Rowhani-Rahbar A, Hawes SE, Sow PS, et al. The impact of HIV status and type on the clearance of human papillomavirus infection among Senegalese women. *J Infect Dis.* 2007;196:887-894.

**29.** Johnson L. *Thembisa version 2.5: A model for evaluating the impact of HIV/AIDS in South Africa.* Cape Town, South Africa: Centre for Infectious Disease Epidemiology and Research, University of Cape Town, 2016.

**30.** Bendavid E, Grant P, Talbot A, et al. Cost-effectiveness of antiretroviral regimens in the World Health Organization's treatment guidelines: a South African analysis. *AIDS.* 2011;25:211-220.

**31.** Cori A, Pickles M, van Sighem A, et al. CD4+ cell dynamics in untreated HIV-1 infection: overall rates, and effects of age, viral load, sex and calendar time. *AIDS.* 2015;29:2435-2446.

**32.** Siedner MJ, Ng CK, Bassett IV, et al. Trends in CD4 count at presentation to care and treatment initiation in sub-Saharan Africa, 2002-2013: a meta-analysis. *Clin Infect Dis.* 2015;60:1120-1127.

**33.** Johnson LF, Keiser O, Fox MP, et al. Life expectancy trends in adults on antiretroviral treatment in South Africa. *AIDS.* 2016;30:2545-2550.

**34.** South African National Department of Health. Uniform Patient Fee Schedule for Paying Patients Attending Public Hospitals: Tariffs 2015.: Government Printing Office; 2015.

**35.** National Health Laboratory Service. State Pricing List. National Health Laboratory Service, 2015.

**36.** South African National Department of Health. Uniform Patient Fee Schedule for Paying Patients Attending Public Hospitals: Tarriffs 2014. Government Printing Office; 2014.

**37.** South African National Department of Health. Annexure L: Medical oncology authorisation form for externally funded patients treated at public hospitals - effective 1 April 2016. Government Printing Office; 2016.

**38.** South African Department of Public Service Administration. Salary scales, with translation keys, for employees on salary levels 1 to 12 and covered by Occupation Specific Dispensations (OSDs): Government Printing Office; 2015.

**39.** South African National Department of Health. Affordable Medicines Directorate. Breast cancer agents - costs. 2017.

**40.** Firnhaber C, Mayisela N, Mao L, et al. Validation of cervical cancer screening methods in HIV positive women from Johannesburg South Africa. *PLoS One.* 2013;8:e53494.

**41.** McDonald AC, Denny L, Wang C, et al. Distribution of high-risk human papillomavirus genotypes among HIV-positive and HIV-negative women with and without cervical intraepithelial neoplasia in South Africa. *PLoS One.* 2012;7:e44332.

**42.** Clifford GM, de Vuyst H, Tenet V, et al. Effect of HIV Infection on Human Papillomavirus Types Causing Invasive Cervical Cancer in Africa. *J Acquir Immune Defic Syndr.* 2016;73:332-339.

**43.** McDonald AC, Tergas AI, Kuhn L, et al. Distribution of Human Papillomavirus Genotypes among HIV-Positive and HIV-Negative Women in Cape Town, South Africa. *Front Oncol.* 2014;4:48.

**44.** National Cancer Registry. *Cancer in South Africa.* 2011. Available at: http://www.nioh.ac.za/assets/files/2011%20NCR%20Full%20Corrected%2003_03_2017.pdf.

**45.** Abraham AG, D'Souza G, Jing Y, et al. Invasive cervical cancer risk among HIV-infected women: a North American multicohort collaboration prospective study. *J Acquir Immune Defic Syndr.* 2013;62:405-413.

**46.** Rohner E, Butikofer, L., Maskew, M., Chen, Y.A., Friedman, R., D'Souza, G., Egger, M., Bohlius, J. Global Burden of Cervical Cancer in HIV-infected Women on Antiretroviral Therapy. *Conference on Retroviruses and Opportunistic Infections*. Boston, MA. 2016.
